# Deciphering the performance of macrophages in tumour microenvironment: a call for precision immunotherapy

**DOI:** 10.1186/s13045-024-01559-0

**Published:** 2024-06-11

**Authors:** Belén Toledo, Linrui Zhu Chen, María Paniagua-Sancho, Juan Antonio Marchal, Macarena Perán, Elisa Giovannetti

**Affiliations:** 1 Department of Health Sciences, University of Jaén, Campus Lagunillas, Jaén, E-23071 Spain; 2 Department of Medical Oncology, Cancer Center Amsterdam, Cancer Biology and Immunology, Amsterdam UMC, VU University, Amsterdam, The Netherlands; 3 Biopathology and Regenerative Medicine Institute (IBIMER), Centre for Biomedical Research (CIBM), University of Granada, Granada, E-18100 Spain; 4 Instituto de Investigación Sanitaria ibs. GRANADA, Hospitales Universitarios de Granada-Universidad de Granada, Granada, E-18071 Spain; 5 Department of Human Anatomy and Embryology, Faculty of Medicine, University of Granada, Granada, E-18016 Spain; 6 Excellence Research Unit “Modeling Nature” (MNat), University of Granada, Granada, E-18016 Spain; 7 Cancer Pharmacology Lab, Fondazione Pisana per la Scienza, San Giuliano, Pisa, 56017 Italy

**Keywords:** Tumour-associated macrophages, Cancer cell, Tumour microenvironment, Polarization, Immunity, Immunotherapy

## Abstract

Macrophages infiltrating tumour tissues or residing in the microenvironment of solid tumours are known as tumour-associated macrophages (TAMs). These specialized immune cells play crucial roles in tumour growth, angiogenesis, immune regulation, metastasis, and chemoresistance. TAMs encompass various subpopulations, primarily classified into M1 and M2 subtypes based on their differentiation and activities. M1 macrophages, characterized by a pro-inflammatory phenotype, exert anti-tumoural effects, while M2 macrophages, with an anti-inflammatory phenotype, function as protumoural regulators. These highly versatile cells respond to stimuli from tumour cells and other constituents within the tumour microenvironment (TME), such as growth factors, cytokines, chemokines, and enzymes. These stimuli induce their polarization towards one phenotype or another, leading to complex interactions with TME components and influencing both pro-tumour and anti-tumour processes.

This review comprehensively and deeply covers the literature on macrophages, their origin and function as well as the intricate interplay between macrophages and the TME, influencing the dual nature of TAMs in promoting both pro- and anti-tumour processes. Moreover, the review delves into the primary pathways implicated in macrophage polarization, examining the diverse stimuli that regulate this process. These stimuli play a crucial role in shaping the phenotype and functions of macrophages. In addition, the advantages and limitations of current macrophage based clinical interventions are reviewed, including enhancing TAM phagocytosis, inducing TAM exhaustion, inhibiting TAM recruitment, and polarizing TAMs towards an M1-like phenotype. In conclusion, while the treatment strategies targeting macrophages in precision medicine show promise, overcoming several obstacles is still necessary to achieve an accessible and efficient immunotherapy.

## Introduction

During the last decades, cancer research has focused on understanding the genetic and molecular features of cancer cells; however, emerging evidence underscores the pivotal role of tumour-associated macrophages (TAMs) and other components of the tumour microenvironment (TME) in cancer progression [1, 2]. Several studies have shown that TAMs can impact tumour response to therapy, promoting resistance to various treatments, including chemotherapy [3, 4].

Macrophages, essential constituents of the immune system’s frontline, showcase a wide array of characteristics pivotal to their role in both health and disease. They perform multifaceted functions in maintaining tissues, combating pathogens, and regulating inflammatory responses [5]. Functioning as professional phagocytes, macrophages play a crucial role in tissue homeostasis and immune surveillance [6]. They demonstrate notable heterogeneity and plasticity within the human immune system, being distributed across various tissues [7]. Through their phagocytic capabilities, macrophages efficiently eliminate invading pathogenic microorganisms, serving as the primary nonspecific defense (innate immunity), and also facilitate the initiation of specific defense mechanisms by enabling antigen processing and presentation (adaptive immunity) [8].

**Fig. 1:**
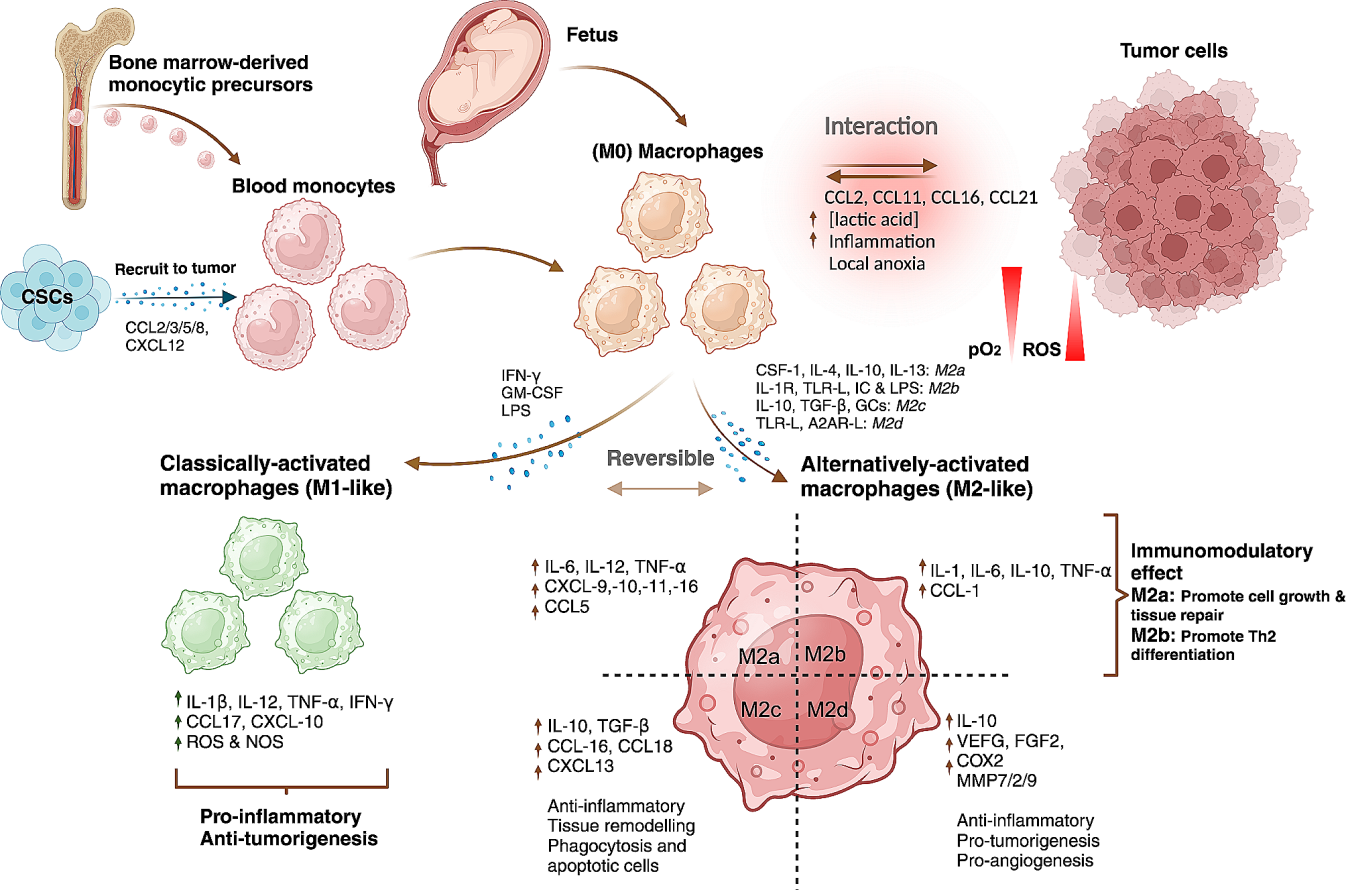
The diverse cellular origins of TAMs, give rise to two primary subpopulations with distinct roles in shaping the immune response. Tissue macrophages typically arise from circulating monocytes originating in the bone marrow, undergoing differentiation into M0 macrophages, which subsequently polarize into M1 or M2 states in response to microenvironmental signals. Notably, macrophages can also originate from embryonic precursors during early fetal development, bypassing monocytic intermediates. Interactions between macrophages and tumour cells begin as early as the M0 stage, influencing subsequent polarization and recruiting additional macrophages to the tumour site via chemoattractants released by both CSCs and tumour cells. The M1 phenotype, primarily induced by factors like IFN-γ, LPS, and GM-CSF, leads to the secretion of pro-inflammatory cytokines such as IL-6, IL-12, IL-23, and TNF-α, contributing to enhanced inflammatory responses and cytotoxic effects on pathogens and tumour cells. In contrast, the M2 phenotype encompasses subtypes like M2a, M2b, M2c, and M2d, each influenced by specific stimuli such as CSF-1, IL-4, IL-13, and IL-10, which are associated with parasite infection, tissue remodelling, allergic diseases, and angiogenesis. Under typical conditions of the TME, characterized by low oxygen levels, high lactic acid levels, inflammation, and oxidative stress, macrophages tend to adopt the M2 phenotype, marked by elevated levels of IL-10, TGF-β, pro-angiogenic factors, and tissue-remodelling enzymes like MMPs. Consequently, this M2 phenotype promotes angiogenesis, immunosuppression, and tumour progression. Abbreviations: C-C motif chemokine ligand, (CCL) Cancer stem cells (CSCs), colony stimulating factor 1 (CSF-1), chemokine C-X-C motif ligand (CXCL), glucocorticoid (GC), granulocyte macrophage colony-stimulating factor (GM-CSF), immune complex (IC), interferon-gamma (IFN-γ), interleukin (IL), IL-1 receptor antagonist (IL-1ra), lipopolysaccharide (LPS), major histocompatibility complex (MHC), matrix metalloproteinases (MMPs), nitric oxide (NO), reactive oxygen species (ROS), tumour-associated macrophages (TAMs), transforming growth factor beta (TGF-β), tumour microenvironment (TME), tumour necrosis factor alpha (TNF⍺), toll-like receptor (TLR)

It is widely acknowledged that the majority of macrophages originate from monocytes in the peripheral blood circulation. However, the precise mechanisms and origin of macrophages remain unclear [9, 10]. Monocytes are mobilized from the bone marrow and travel through the bloodstream to tissues and organs, where they proliferate and differentiate into tissue-specific macrophages. Nevertheless, certain tissue-resident macrophages, such as Kupffer cells in the liver and alveolar macrophages in the lungs, do not originate from blood monocytes, and the mechanisms governing their genesis, self-renewal, proliferation, and replacement remain elusive [11] (Fig. 1). Dick and colleagues have demonstrated the coexistence of blood monocyte-derived macrophages and tissue-resident macrophages that proliferate in situ in various tissues, including the brain, spleen, and lung highlighting the different phenotypes and roles of these macrophages [12].

Macrophages play a dual role in the immune response. They induce inflammation and secrete various signalling cytokines to facilitate tissue repair. However, they can also have detrimental effects, particularly in the context of autoimmunity and cancer progression [13].

**Fig. 2:**
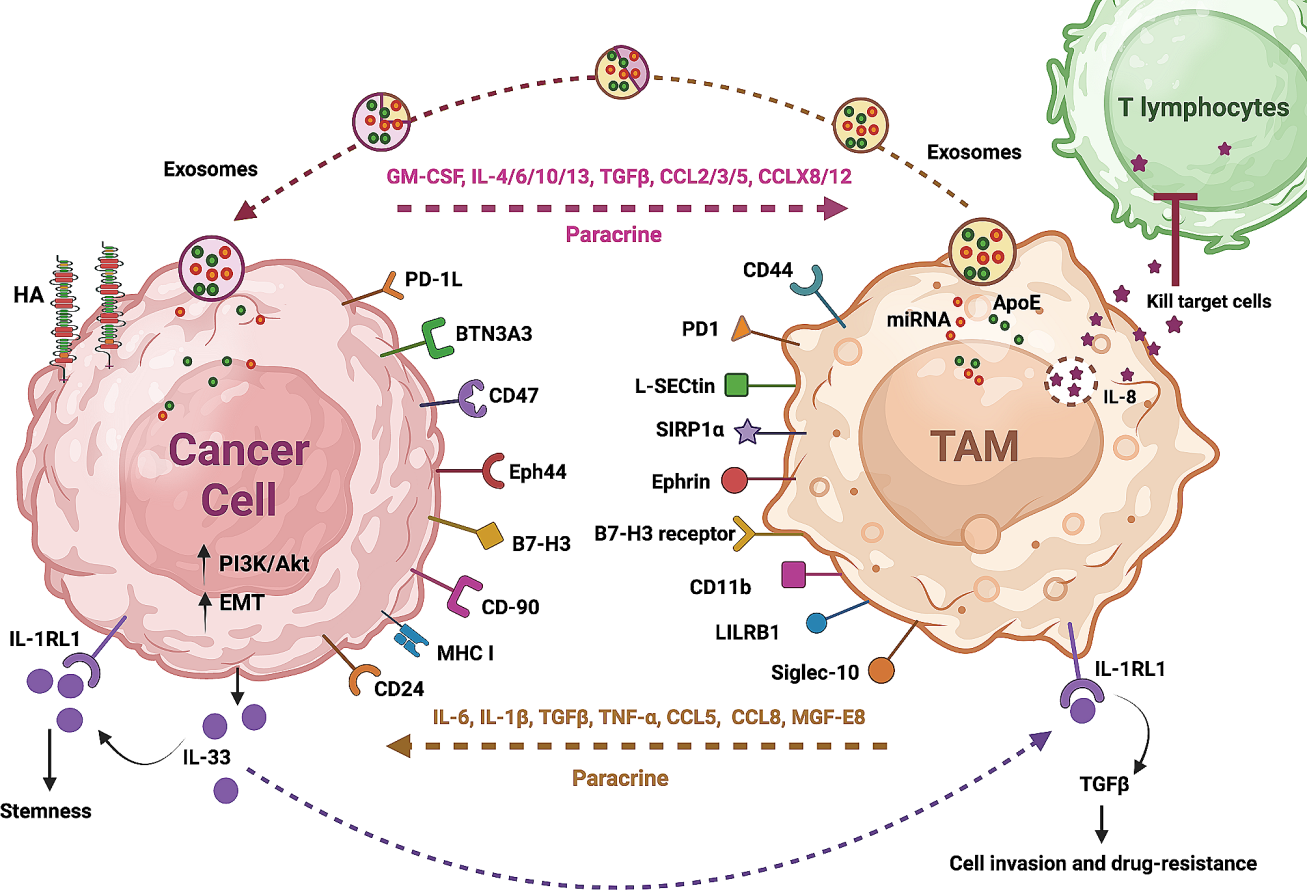
Crosstalk between tumour cells and tumour-associated macrophages (TAMs). TAMs secrete chemokines and cytokines such as IL-6, IL-8, and IL-10, which actively contribute to cancer advancement. Notably, IL-8 released by TAMs exerts cytotoxic effects on T lymphocytes. Additionally, various juxtacrine interactions between tumour cells and TAMs play a pivotal role in inducing immunosuppression. The PD-1/L1 signalling pathway further exacerbates tumour immune evasion by impeding the normal functioning of macrophages and other immune effector cells. Furthermore, the interaction of B7-H3 with its receptor has been implicated in the inhibition of T lymphocytes, thus facilitating tumour immune evasion. The SIRPα/CD47 and CD24/Siglec-10 pathways serve as the “do-not-eat-me” signal, wherein tumour cells over-expressing CD47 and CD24 are recognized as self-normal cells, thereby evading phagocytosis by macrophages. Another significant mechanism of tumour evasion involves LILRB1/MHC class I component β2-microglobulin, which inhibits the phagocytosis of tumour cells by macrophages. Exosomes facilitate intercellular communication by transporting various molecules, including exosomal mRNA, circRNA, lncRNA, miRNA, lipids, and proteins. Interestingly, exosomes exhibit dynamic alterations in their cargo during transit from the origin to the destination cell. ApoE is highly expressed in TAMs and is transferred, along with other molecules, via exosomes to cancer cells, activating the PI3K-Akt signalling pathway and promoting cytoskeletal remodeling, EMT, and cancer cell migration. Other juxtacrine mechanisms, such as Eph44-ephrin interaction, regulate immune cell processes, including proliferation, survival, apoptosis, activation, and migration. CD44, a transmembrane adhesion molecule, plays a crucial role in binding and metabolizing hyaluronic acid (HA) and serves as an effective phagocytic receptor, influencing inflammation, phagocytosis, and multi-drug resistance. Interactions such as CD44-HA, BTN3N3–L-SECtin, CD90-CD11b, and Eph44-ephrin also also trigger signals that support the maintenance of cancer stem cells. Furthermore, IL-33 released by tumour cells sustains stemness via autocrine interaction with IL-1RL1, while also promoting tumour cell invasion and drug resistance through TAM-mediated TGF-β release and TAM proliferation and differentiation in a paracrine manner. This intricate interplay results in the amplification of the aforementioned crosstalk. Abbreviations: Apolipoprotein E (ApoE), C-C motif chemokine Ligand, (CCL), circular RNA (circRNA), epithelial-mesenchymal transition (EMT), granulocyte-macrophage colony-stimulating factor (GM-CSF), hyaluronic acid (HA), interleukin (IL), Janus kinase (JAK), long non-coding RNA (lncRNA), macrophage colony-stimulating factor (M-CSF), milk fat globule-EGF factor 8 (MGF-E8), major histocompatibility complex (MHC), microRNA (miRNA), messenger RNA (mRNA), programmed death-ligand 1 (PD-L1), sialic acid binding Ig-like lectin (Siglec), signal transducer and activator of transcription 3 (STAT3), tumour-associated macrophages (TAMs), transforming growth factor beta (TGF-β), tumour necrosis factor alpha (TNF⍺)

TAMs are recruited to the TME during the initial stages of tumour development by cytokines and chemokines released by cancer cells [14] (Fig. 2). Upon arrival, these cells start an interaction with tumour cells through complex signalling networks, influencing tumour growth, invasion, and metastasis, and other cancer hallmarks [15]. The behaviour of TAMs at different tumour stages depends on their diverse origins, the influence of the tissue environment, and the activation of specific intracellular regulatory signals. Through a process known as “macrophage polarization,” TAMs can differentiate into two subtypes: M1 or M2-like TAMs [16, 17]. The M1 subtype is classically activated, while the M2 subtype is alternatively activated, depending on the signals received from their microenvironment (Fig. 1).

Despite intriguing findings showing that TAMs influence tumour progression, metastasis, therapeutic resistance, and immune responses, there are still many unsolved issues about the connection between TAMs and the tumour. Are TAMs initially physiological barriers combating the onset of tumour mass aggression? Or do they align with the tumour even before tumour cells initiate proliferation? Alternatively, do macrophages only join the battle against cancer upon recruitment by tumour cells?

Here, we will review the latest findings about the interplay between macrophages and the TME and carcinogenic events thereafter, with the aim of discussing novel therapeutic approaches targeting this interplay as means to fight cancer.

## The interaction between TAMs and the TME and its implication for cancer prognosis

The TME is a complex ecosystem consisting of various cell types, including stromal cells, cancer cells and immune cells such as dendritic cells (DCs), natural killer cells (NK), myeloid-derived suppressor cells (MDSCs), mast cells, tumour-associated neutrophils (TANs) and recruited macrophages [18].

The recruitment of TAMs to the TME is a complex interplay involving various cellular components and surrounding conditions. This process primarily relies on juxtacrine and paracrine signalling mechanisms, with cytokines and exosome secretion playing significant roles. A chemokine released by tumours that plays a key role in TAM recruitment is granulocyte-macrophage colony-stimulating factor (GM-CSF). Studies have demonstrated that at low circulating levels, this factor exhibits anti-tumourigenic effects by activating dendritic cells (DCs) within tumours. However, in advanced stages of cancer, characterized by high levels of GM-CSF, it switches to recruit TAMs and promotes oncogenesis. In fact, overexpression of this cytokine, as well as CSF, has been observed in various cancer types, including breast cancer, colon cancer, and cholangiocarcinoma, resulting in enhanced recruitment and infiltration of TAMs to the tumour site [19–21]. Additionally, CSF has been found to induce the production of IL-8 from TAMs in colon cancer cells. Subsequently, interleukin-8 (IL-8) activates the protein kinase C signalling pathway in colon cancer cells, leading to further release of CSF by the tumour, thereby attracting more TAMs [20]. This illustrates how the crosstalk between TAMs and cancer cells forms a vicious cycle (Fig. 2). Other noteworthy tumour-released cytokines that induce TAM recruitment include IL-17, IL-34, and CSF-2, observed in lung cancer, osteosarcoma, and breast cancer, respectively [22–24]. Apart from cytokines, certain tumour-released chemokines also play a significant role in TAM recruitment. Major examples include C-C motif chemokine ligand 2 (CCL2), CCL5, CCL20, C-X-C motif chemokine ligand 4 (CXCL4) and CXCL12, implicated in bladder cancer, colon cancer, breast cancer, and non-small cell lung cancer (NSCLC), respectively [25–28]. Ultimately, the dependence on these tumour-released factors underscores the critical role of an inflammatory TME in facilitating the essential recruitment of TAMs, which become accomplices in tumourigenesis.

Interestingly, tumour cells are not the sole initiators of TAM recruitment; other stromal cellular components of the TME are also actively involved in this process. In hepatocellular carcinoma, TANs recruited to the tumour site under the influence of tumour-derived CXCL5 release CCL2 and CCL17, which subsequently attract TAMs and contribute to cancer progression [29]. Additionally, cancer-associated adipocytes collaborate with breast cancer cells to release CCL2, along with adipokines such as leptin and lauric acid, collectively recruiting TAMs into the TME [30, 31]. Furthermore, mesenchymal stromal cells and cancer-associated fibroblasts (CAFs) promote an inflammatory TME conducive to TAM recruitment in a C-C chemokine receptor type 2 (CCR2)-dependent manner and an IL-8/C-X-C chemokine receptor type 2 (CXCR2)-dependent manner, respectively [32, 33].

Another molecular mechanism that modulates the interaction between tumour cells and TAMs involves a membrane protein called cluster of differentiation 47 (CD47). It is present on the membrane surfaces of various cell types, including tumour cells. Signal regulatory protein alpha (SIRPα), which is the ligand of CD47 is a membrane protein that is primarily expressed by bone marrow cells and TAMs (Fig. 2). Collectively, these cells form a typical immunoreceptor tyrosine-based inhibitory motif (ITIM). The cytosolic tyrosine phosphatase SHP-1 or SHP-2 can be recruited and activated through the interaction of the NH2 terminal domain of the ITIM motif with the single domain of CD47. Consequently, this interaction can limit TAMs’ phagocytosis of cancer cells by dephosphorylating numerous substrates and regulating downstream signalling cascades. As a result, CD47 is sometimes referred to as the “do-not-eat-me” signal [34].

Macrophage-mediated programmed cell removal (PrCR) is crucial for monitoring and eradicating tumours. The activation of toll-like receptor (TLR) pathways in macrophages initiates the Btk signalling pathway, resulting in the phosphorylation and dissociation of the cell surface calreticulin (CRT) in the endoplasmic reticulum [35]. In order to target cancer cells for phagocytosis, the dissociated CRT is expressed on the surface of macrophages and subsequently forms the CRT/CD91/C1q complexes [36]. “Do-not-eat-me” signals counteract the production of PrCR on tumour cells via binding to SIRPα on macrophages, thus preventing phagocytosis. Conversely, “do-not-eat-me” cues can be prevented by blocking CD47 on tumour cells. Therefore, to improve PrCR, activation of the TLR signalling pathway in macrophages can work in conjunction with the inhibition of CD47 on tumour cells. Some studies propose that, even following CD47 blockade, tumour cells may still evade macrophage phagocytosis. Specifically, research by Weissman and colleagues revealed that tumour cells resist macrophage phagocytosis via an alternative recognition mechanism between tumour cells and macrophages [37]. The signalling protein β2-microglobulin, found on the surface of tumour cells as part of major histocompatibility complex class I (MHC-I), can be targeted for inhibition or downregulation to activate macrophages in vivo, thereby enhancing phagocytosis and eliminating tumour cells. This intervention has been shown to increase the lifespan of tumour-bearing animals by 70% [37] (Fig. 2).

TAMs’ status has also been shown to be strongly linked to cancer stage and prognosis. In general, early-stage tumours are associated with a predominance of anti-tumour TAMs, while advanced-stage tumours are associated with a predominance of pro-tumour TAMs. Therefore, the ratio of pro-tumour to anti-tumour TAMs (also known as the TAM polarization index) has been proposed as a prognostic marker and therapeutic target in cancer [38, 39]. Väyrynen and colleagues demonstrated that a high M1:M2 density ratio in tumour stroma was associated with better cancer-specific survival after the evaluation of the polarization spectrum of TAMs in 931 colorectal carcinomas [40]. However, the localization of these cells has been shown to hold equal or even greater prognostic significance than simply assessing the presence of TAMs in the TME [41].

Precise percentage figures can vary widely depending on the context and methodology used to study TAMs within the TME [42]. What is well-established is that their presence is linked with poor prognosis in many types of cancer, including breast cancer, ovarian cancer, pancreatic cancer, lung cancer, and melanoma, among others [43–46]. In pancreatic cancer, the density of TAMs, in particular those that are CD163 + and CD204+, is associated with advanced disease stage, increased vascularization, and reduced survival [47]. In breast cancer, high levels of TAM infiltration are associated with poor prognosis and increased risk of relapse [48]. Jeong and colleagues concluded that the infiltration of CD163 + M2-like macrophages in tumour nests was associated with larger tumour sizes and unfavourable prognosis for invasive breast cancer (IBC) patients, and served as an independent prognostic marker for reduced overall survival (OS) as well as disease-free survival (DFS) [49]. This is largely attributable to TAMs’ roles in promoting tumour invasion and metastasis by secreting matrix metalloproteinases (MMPs) and other proteases as established in previous studies [50]. On the other hand, infiltration of CD11c + M1-like macrophages in tumour stroma was shown to be prognostic of increased OS and DFS in IBC patients [49]. Similarly, Mei and colleagues reported in their systematic review and meta-analyses that a high density of M1-like TAMs in the tumour islet was indicative of better OS in NSCLC patients, as compared to high density of M2-like TAMs in the tumour stroma which was associated with worse OS in NSCLC patients [44]. And interestingly, the density of total CD68 + TAMs in either tumour islet or stroma was not correlated to patients’ OS [44]. These findings once more reinforce the idea that the spatial distribution of M1- and M2-like TAMs is more reflective of disease prognosis than solely assessing the presence of different TAM phenotypes in the TME.

The complex plasticity and context-dependent nature of TAM functions suggests that targeting TAMs for cancer therapy requires careful consideration of the specific TAM phenotype and its interactions with the TME [51, 52]. Presently, several approaches are being explored to target TAMs as a target in cancer therapy, including reprogramming TAMs to an anti-tumourigenic phenotype, inhibiting their recruitment to the tumour site, and enhancing the anti-tumour immune response [53–55]. However, further research is needed to unravel the complex interplay between macrophages and the TME [56–58].

## Regulation of macrophage polarization

Upon infiltrating peripheral tissues, macrophage’s activation pattern depends on signals received from the local microenvironment, prompting them to adopt different states or functional roles in response to the stimuli they encounter [59].

Naïve macrophages (M0) recognize pathogens, execute phagocytic functions, and upon activation, swiftly polarize into either pro- or anti-inflammatory macrophages to acquire their complete array of functions [60] (Fig. 1). Polarization is a dynamic process that involves the activation of specific signalling pathways and gene expression programs, resulting in distinct functional phenotypes [61]. As mentioned earlier, the M1 phenotype, or activated macrophages, are characterized by the production of proinflammatory cytokines, conversely, the M2 phenotype is associated with the secretion of anti-inflammatory cytokines [62–65].

M1-like macrophages are typically activated by pro-inflammatory stimuli such as LPS from the outer membrane of bacteria, GM-CSF secreted by other immune cells or IFN-γ [66–68] (Table 1). These types of macrophages are characterized by their increased capacity to present antigens and to produce elevated levels of pro-inflammatory cytokines and chemokines, such as IL-1α, IL-1β, IL-6, IL-12, IL-23, as well as C-C motif CCL5, C-X-C motif chemokine factor ligand 9 (CXCL9), CXCL10, cyclooxygenase-2 (COX-2) and TNF-α, which induce positive feedback on non-polarized macrophages, causing them to enter the M1 state [62, 69–71]. In addition to inducing polarization, the chemokines CCL5, CXCL9, and CXCL10 recruit activated T cells, NK cells, and neutrophils to fight infection [72, 73] (Table 2). These features support the role of this phenotype in the defense and initiation of immune responses against pathogens and tumour cells [74]. M1-like macrophages express a large number of surface markers such as Toll-like receptors (TLR) 2 and 4, CD40+, CD68+, CD80+, CD86+, IL-1R+, major histocompatibility complex (MHC)-II, and enzymes such as inducible nitric oxide synthase (iNOS) [75–77]. High expression of iNOS contributes to the secretion of reactive nitrogen species such as NO by these macrophages [78], which are also important factors for anti-infection immunity. They are also known for the secretion of reactive oxygen species (ROS) [79].

**Table 1:** Factors influencing the polarization of macrophages into M1- or M2-like phenotypes

Factors released by tumor/immune cells/pathogens	Mode of Action - Polarization to M1- or M2-like phenotype	References	
**Cytokines/Chemokines**			
GM-CSF	M1-like	[63, 169–172]	
TNF-α			
IFN-γ			
IL-1/-12			
TLR-agonists (i.e. LPS) ^a^			
PAMPs/DAMPs			
IL-4/-13	M2a	[63, 170, 172–177]	
CSF1			
PPAR-γ			
IL-1β/-34	M2b	[63, 105, 168, 170, 172, 174, 178, 179]	
IC + FcγRs			
TLR-agonists (i.e. LPS)			
IL-10	M2c	[63, 168, 170, 172, 174, 180, 181]	
TGF-β			
Glucocorticoids			
PGE2			
IL-6/-10	M2d	[168, 170, 172, 174, 182, 183]	
TNF-α			
A2R-agonists (i.e. LIF)			
TLR-agonists (i.e. LPS)			
LPS			
**miRNAs**			
miR-21	M1-like	[135, 170, 184–194]	
miR-125b			
miR-127			
miR-155			
miR-181			
miR-451			
miR-495			
miR-720			
		[135, 170, 184–186, 188, 189, 195–197]	
miR-92a	M2-like		
miR-124			
miR-125a/b			
miR-130b-3p			
miR-145-5p			
miR-146a			
miR-511-5p/3p			
**TME status**			
Local anoxia	M2-like	[63, 132, 198]	
High lactic acid concentration			
High ROS			

*Abbreviations* A2R, adenosine receptor 2; CSF-1, colony stimulating factor 1; DAMP, damage-associated molecular pattern; FcγR, Fragment crystallizable region γ receptors; G-CSF, granulocyte colony-stimulating factor; GM-CSF, granulocyte-macrophage colony-stimulating factor; IC, immune complex; IFN-γ, interferon gamma; LIF, leukemia inhibitory factor; LPS, lipopolysaccharide; M-CSF, macrophage colony-stimulating factor; PAMP, pathogen-associated molecular pattern; PGE2, prostaglandin E2; PPAR-γ, peroxisome proliferator-activated receptor gamma; ROS, reactive oxygen species; TGF-β, transforming growth factor-beta; TLR, toll-like receptor; TNF-α, tumor necrosis factor alpha

^a^ from microbes

**Table 2:** Effects of factors secreted by macrophage subtypes on the TME

Macrophage subtypes	Factors released by M1-/M2-like macrophages	Influence on TME	References
M1-like	TNF-α ^high^	Induces inflammatory response. Attract monocytes and M0 macrophages and induce Th1-response	[50, 88, 132, 87, 101, 168, 170, 183, 270–274]
	IL-1β/-6/-10 ^low^/-12 ^high^/-23 ^high^		
	CXCL1-3/5/8–10 ^high^/11	Attract monocytes and M0 macrophages	
	CCL2/5/8		
	IFN-γ		
	ROS	Anti-infection/-pathogen immunity	
	iNOS		
M2a	IL-1ra ^high^	Induce Th2 response, tissue remodeling, wound healing, monocyte recruitment, tumor progression, debris removal	[3, 87, 101, 102, 108, 135, 170, 179, 182, 271–275]
	IL-4 ^high^/-10 ^high^/ -12 ^low^/-13/-23 ^low^		
	CXCL9/11/16		
	CCL17/18/22		
	Arg-1		
	Chi3l3 ^b^		
	FIZZ1		
	TGF-β		
M2b	IL-1β ^low^/-6/-10 ^high^/-12 ^low^	Immunoregulation, tumor progression, induce Th2 response, recruit Th2 and Treg cells, anti-pathogen and parasite immunity	
	TNF-α		
	CCL1		
	CXCL1/3		
M2c	IL-10 ^high^	Immune-suppression, tumor progression, matrix remodeling, tissue remodeling, regulatory function	
	TGF-β		
	Arg-1		
	CXCL13		
	CCL16/18		
M2d	IL-10 ^high^/-12 ^low^	Immune-suppression, induce angiogenesis, tumor progression, monocyte recruitment to tumor site, tumor invasion and metastasis, ECM remodeling/degradation, intra-/extravasation	
	iNOS ^high^		
	TNF-α ^low^		
	TGF-β ^high^		
	VEGF ^high^		
	Arg-1		
	COX2		
	MMP7/2/9 ^high^		
	iNOS ^b^		
	Serine proteases		
	Cathepsins		
	CCL1 ^low^/17 ^low^/18 ^high^/22 ^low^		
	CXCL10/16		
	PTX3 ^low^		

*Abbreviations* ADO, adenosine; CCL, Chemokine (C-C motif) ligand; Chi3l3, Chitinase-3-like protein-3; COX2, Cyclooxygenase-2; CSF-1, colony stimulating factor 1; CXCL, chemokine (C-X-C motif) ligand; FGF2, Fibroblast Growth Factor 2; FIZZ1, found in inflammatory zone 1; G-CSF, granulocyte colony-stimulating factor; GM-CSF, granulocyte-macrophage colony-stimulating factor; IC, immune complex; IDO, Indoleamine 2,3-Dioxygenase 1; IFN-γ, interferon gamma; iNOS, Inducible Nitric Oxide Synthase; LPS, lipopolysaccharide; M-CSF, macrophage colony-stimulating factor; MMP, Matrix metalloproteinase; PGE2, prostaglandin E2; PPAR-γ, peroxisome proliferator-activated receptor gamma; PTX-3, pentraxin-3; ROS, reactive oxygen species; TGF-β, transforming growth factor-beta; TLR, toll-like receptor; TNF-α, tumor necrosis factor alpha; VEGF, Vascular endothelial growth factor

^b^ In mouse

In some cases, these conditions created by M1 can also generate ideal conditions for a pro-inflammatory microenvironment, thereby potentially exerting pro-tumour effects, such as in pancreatic ductal adenocarcinoma (PDAC) [80]. Furthermore, they can also promote tumour growth and metastasis by producing MMPs which degrade the ECM, facilitating the invasion of cancer cells, and contributing to angiogenesis by producing VEGF [81, 82]. However, in most types of cancers, M1 macrophages have been shown to have anti-tumour activity by promoting cancer cell death and enhancing tumour immunity [83–85].

On the other hand, M2-like macrophages, also known as alternatively activated macrophages, are induced by anti-inflammatory stimuli, such as colony stimulating factor-1 (CSF-1), IL-4, IL-13, and IL-10 (Table 1). This cellular subtype produces anti-inflammatory cytokines, such as IL-10, glucocorticoids, TLR-associated ligands and TGF-β which are associated with their various functions [86, 87] (Table 2). It is known that they are capable of recruiting neutrophils and Th2 cells by secreting the chemokine CCL17/18, playing an important role in homeostasis [88]. Additionally, they can participate in phagocytic activity in parasite infection and in resolving inflammation in diseases such as allergies. Moreover, they may contribute to angiogenesis and tissue repair and remodelling [69].

These TAMs possess the capability to suppress the immune response against tumours through various mechanisms, for instance, by secreting the cytokine CCL22 to recruit regulatory T cells (Tregs) (discussed further in Sect. 4.5). Additionally, the IL-10 and prostaglandin E2 (PGE2) that they release inhibit the activation and proliferation of T cells. They can also metabolize L-arginine, an essential amino acid for T and NK cells, through the enzyme Arginase-1 (Arg-1), and produce oxygen radicals and nitrogen species, which are detrimental to these cells, thereby inhibiting their antitumour activity. These processes have been linked to the development of therapeutic resistance to chemotherapy and radiotherapy in colorectal, pancreatic, or ovarian cancers [4, 52, 89–92]. However, this immune cell subtype can also have beneficial effects in cancer therapy, as it has been observed that they can induce an anti-inflammatory response and promote tissue repair during cancer treatment [93, 94].

Some studies have categorized M2-like macrophages into 4 subsets, namely M2a, M2b, M2c, M2d, each characterized by different stimuli and functions [95–97] (as described in Tables 1 and 2). M2a are primarily activated by the presence of IL-4 and IL-13, and they predominantly play an immunomodulatory role. They promote tissue repair, facilitate cell growth and participate in phagocytosis of apoptotic cells. Particularly, M2a macrophages express high levels of mannose receptors, CD206 which are associated with angiogenesis [98], metastasis, as seen in lung metastasis from pancreatic ductal adenocarcinoma [99], and tumour recurrence [100–102]. Meanwhile, M2b macrophages are activated by immune complexes, as well as IL-1β and TLR activation by agonists like LPS. This subset is renowned for its immunoregulatory function, playing a crucial role inducing TH2 cell responses [103], which are essential for immunity against pathogens and parasites [104]. This population of macrophages, which express high levels of CD86 [105], has also been associated with resistance to bevacizumab in triple-negative breast cancer [106] and implicated in fostering an immunosuppressive environment in colorectal cancer [107]. The M2c subtypes respond to glucocorticoids and immunosuppressive cytokines such as IL-10 and TGF-β, and are characterized by high expression of the scavenger receptor CD163 on its surface [108, 109]. This subset is primarily involved in immunosuppression, as they release a range of cytokines for the inflammation resolution, allowing tumour proliferation as demonstrated in glioma and melanoma [110, 111]. Finally, M2d macrophages are distinguished by their response to adenosine receptor agonists and TLR2, TLR4, TLR7, and TLR9 agonists [112]. This subset supports various processes such as angiogenesis mediated by VEGF [112], immunosuppression via IL-10 secretion as has been seen in ovarian cancer [113], and tissue invasion by releasing ECM degradation factors such as proteolytic enzymes including MMPs, cathepsins and serine proteases [114–117]. These mechanisms collectively promote tumour growth and invasion [101, 108]. Wyckoff and colleagues supported growing evidence from in vivo analyses supporting the view that the motility of metastatic cancer cells and their egress into the circulation occurs in close cooperation with tumour-associated macrophages [118].

In physiological conditions, the regulation of the balance between M1 and M2 macrophages is paramount for maintaining tissue homeostasis and an appropriate immune response [119–121]. However, dysregulation of macrophage polarization contributes to the development of various diseases, including cancer. For instance, in autoimmune diseases such as rheumatoid arthritis, macrophages can be polarized towards both forms, thus contributing to disease pathogenesis. Similarly, in chronic inflammatory conditions like atherosclerosis, they may adopt a more pro-inflammatory phenotype, exacerbating inflammation and disease progression [122, 123]. In cancer, studies have demonstrated that the population of TAMs is in a constant state of transition between these two phenotypes, and their polarization is tightly regulated by the type and concentration of different signals within the TME [124, 125]. Roca and colleagues demonstrated that by increasing CCL2 or IL-6 stimulation, M0 macrophages developed a polarization toward the CD206 + M2-type phenotype [126].

**Fig. 3:**
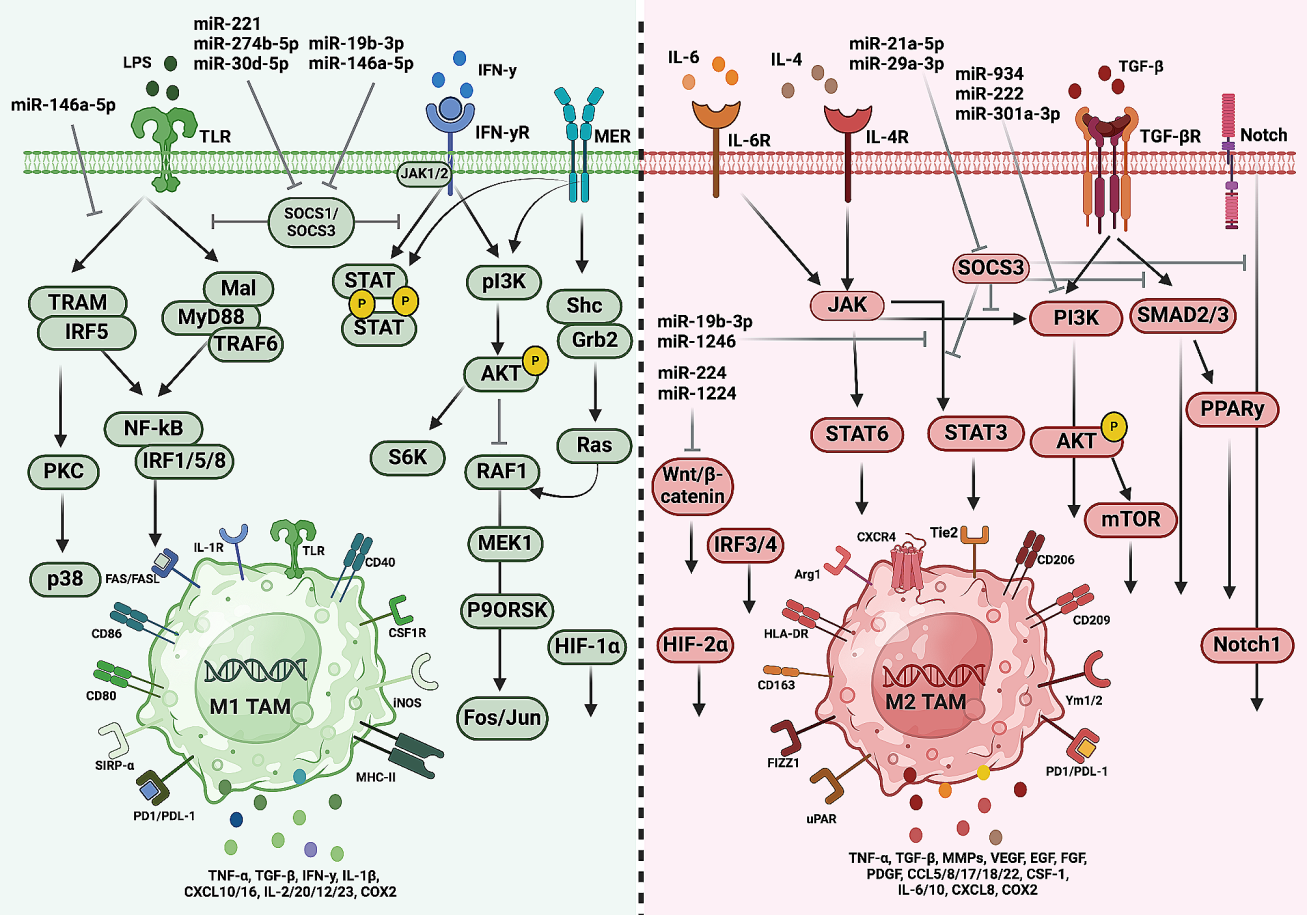
Principal pathways implicated in the activation of tumour-associated macrophages (TAMs). Macrophages of the M1 phenotype express innate immune receptors like Toll-like receptors (TLRs) during their development and maturation, enabling recognition of pathogen-associated molecular patterns such as LPS from microbial surfaces. Ligand binding to TLR4 by LPS activates downstream signalling pathways, including the MyD88-dependent pathway or the IRF5-dependent pathway, leading to further signalling via the NF-κB pathway or the p38-MAPK pathway, respectively. These pathways collectively promote the expression of inflammatory factors and polarization of M1 macrophages. Additionally, polarization towards M1 can be induced by IFN-y binding to its receptor, activating the JAK/STAT1 and PI3K/AKT/Fos/Jun pathways. The latter is also activated upon ligand binding of receptor tyrosine kinases like MER. Inhibitors such as SOCS1/3 can inhibit both the TLR4/MyD88/NF-κB and JAK/STAT1 pathways, thus hindering M1 polarization activity by blocking upstream signalling of these pathways. Conversely, M2 polarization primarily occurs through the interaction of IL-4/6 with their receptors, activating the JAK/STAT3/6 signalling pathway. Moreover, activation of the TGF-βR results in downstream signalling via the PI3K/Akt/mTOR and TGF-βR/Smad/PPARy pathways. Additionally, activation of the Wnt/β-catenin signalling pathway by tumour-derived Wnt ligands stimulates M2 polarization. Notch signalling also contributes to M2 polarization through a positive feed-forward loop, promoting production of IL-6, IL-10, and IL-12. Inhibition of these pathways by SOCS3 prevents M2 polarization. Certain exosomal miRNAs regulate macrophage polarization by affecting the mentioned signal pathways or transcription factors. Furthermore, detection of oxidative stress leads to upregulation of HIF-1α and HIF-2α, with HIF-1α favoring M1 polarization and HIF-2α promoting M2 polarization. Abbreviations: protein kinase B (AKT), hypoxia-inducible factors (HIF), interferon regulatory factors (IRF), mitogen-activated protein kinase kinase (MEK), mammalian target of rapamycin (mTOR), bone marrow differentiation factor 88 (MyD88), nuclear factor kappa B (NF-κB), LPS Phosphatidylinositide 3-kinases (PI3K), protein kinase C (PKC), peroxisome proliferator-activated receptor (PPAR), rapidly accelerated fibrosarcoma (RAF), suppressors of cytokine signalling (SOCS), signal transducer and activator of transcription (STAT), Toll-like receptor (TLR), TNF receptor associated factors (TRAF)

Other mechanisms, including cellular signalling, post-transcriptional regulation, epigenetic changes, and cellular metabolism can also significantly influence the phenotypic and functional directionality of these immune cells. For instance, the Notch receptor and the peroxisome proliferator-activated receptor (PPAR), along with transcription factors such as nuclear factor kappa B (NF-κB), hypoxia-inducible factor (HIF), or interferon regulatory factor (IRF), as well as signalling pathways such as phosphatidylinositol 3-kinase (PI3K)/protein kinase B (AKT), TGF-β/Smad, or Janus kinase/signal transducer and activator of transcription (JAK/STAT) also influence this process [127, 128]. Furthermore, post-transcriptional regulation through non-coding RNAs (ncRNAs) such as microRNAs (miRNAs), also plays a prominent role in this regulation [63]. These microRNAs, frequently transported by exosomes, can influence the gene expression of these cells, alongside epigenetic modifications such as DNA methylation and histone modifications [129] (Fig. 3). Binenbaum and colleagues found that miR-365 was amongst one of the most differentially upregulated microRNAs in M2-like macrophages compared to M1-like macrophages. It was also found that the macrophage-derived exosomal transfer of miR-365 significantly mediated the sensitivity of PDAC cells to gemcitabine by inducing resistance [130]. Moreover, a recent study showed that the combination of the DNA methylation inhibitor 5-Aza-2’-deoxycytidine (5-Aza-dC) and the histone deacetylation inhibitor protomycin A were capable of reprogramming M2-like macrophages into M1-like macrophages, and resulted in a decrease of M2-related cytokines while an increase in M1-related ones. Furthermore, it was reported that the microRNA, miR-7083-5p, was a key modulator in this phenotype skewing as seen by its upregulation in M2-like macrophages after treatment, and its effect on inducing the M1-like phenotype after transfecting M2-like macrophages with this microRNA [131].

Additionally, recent research has also unveiled the relationship between autophagy and cell metabolism in regulating this process [89, 132]. Given that an imbalance in macrophage polarization has been identified as a potential necessary factor in cancer development, it is essential to delve deeper into understanding the underlying mechanisms of this process. Fully comprehending these mechanisms not only provides insights into the pathophysiology of immune-related disorders but also opens the door to targeted therapies, particularly aimed at TAMs.

### Signal pathways, transcription factors and post-transcriptional regulation

Macrophage plasticity is largely supported through the relay of stimulus from the TME to the nuclear compartments of these cells, via membrane receptors and a variety of signalling pathways that collectively lead to the reprogramming of gene expression in macrophages [133]. This ultimately dictates the phenotypic outcome of macrophages. Specific signalling pathways, from upstream receptors to downstream targets, characteristic of M1 or M2-like phenotypes, are shown in Fig. 3.

The differentiation toward M1-like macrophages is induced through various mechanisms: Firstly, activation of TLRs by binding of pathogen-associated molecular patterns (PAMPs) ligands, such as LPS. This interaction initiates a signalling cascade within the cell that leads to the activation of the TLR4/NF-κB signalling pathway, implicated in anti-pathogen immunity [133]. The NF-κB protein can be stimulated through the bone marrow differentiation factor 88 (MyD88) or via interferon regulatory factor 5 IRF5. Once this transcription factor is activated, it translocates to the cell nucleus and promotes the transcription of genes involved in a proinflammatory immune response and defence against pathogens. Inhibition of this signalling cascade has been shown to inhibit M1 polarization activity [134]. Secondly, the interaction of IFN-γ with its receptors present on the surface membrane of macrophages. This binding activates the JAK/STAT pathway downstream, leading to phosphorylation and activation of STAT1 that translocates to the cell nucleus. There, it acts as a transcription factor and regulates the expression of specific genes involved in the production of proinflammatory cytokines, antigen presentation, and stimulation of the immune response, associated with the function of M1 macrophages [135]. This pathway is negatively regulated by the suppressor of cytokine signalling (SOCS), so its depletion by some miRNAs results in an increase in M1 macrophages [136]. It has been observed that factors such as TNF-α and HIF-1α favourably intervene in M1 polarization [133, 135].

In the case of M2-like macrophages, several important pathways shift the differentiation balance towards this phenotype. The binding of TGF-β to its type I and II receptors on macrophages initiates two crucial signalling pathways: PI3K/Akt/mTOR and TGF-β/Smad. PI3K activates Akt through the generation of PIP3, subsequently triggering mTOR activation in the cytoplasm. This protein kinase is fundamental in protein translation and in the regulation of transcription factors and miRNAs. In the TGF-β/Smad pathway, Smad2/3 are activated by phosphorylation and subsequently translocate to the cell nucleus to regulate gene expression. Both pathways are closely interconnected and cooperate to induce the polarization of macrophages towards the M2 phenotype [133, 135]. On the other hand, the binding of IL-4 and IL-6 to their receptors on these cells, induces downstream signalling of the JAK/STAT pathway. Ultimately, this leads to the activation of STAT3 and STAT6 which induces the expression of M2 target genes. Likewise, the interaction of Notch ligands with their receptors on the surface of macrophages activates the Notch pathway, which also induces the expression of genes related to this phenotype, such as Arg-1 and IL-10. Additionally, it is known that the Wnt/β-catenin signalling pathway plays a role in this process. Its activation leads to the stabilization and nuclear translocation of β-catenin, which modulates the expression of genes associated with inflammation resolution and tissue homeostasis. Hypoxia-inducible factor-2α (HIF-2α) is involved in both the Notch and Wnt/β-catenin pathways. Under hypoxic conditions, this factor can modulate the expression of key genes implicated in the anti-inflammatory phenotype [133, 135]. The collective downstream activation of the mentioned pathways results in the expression and release of immunosuppressive, pro-angiogenic and tissue remodelling factors that define the pro-oncogenic traits of M2-like macrophages.

As it was mentioned, it is important to also report the potential role of certain miRNAs in macrophage polarization. In fact, small non-coding RNA molecules, transmitted through exosomes, released into the TME by immune and tumour cells, might exert regulatory control by modulating key signalling pathways and transcription factors [135]. For instance, miR-30d-5p plays a role in the NF-κB signalling pathway promoting M1 polarization by inhibition of SOCS1, which acts as a negative regulator of this signalling pathway. Administering inhibitors of this miRNA can counteract these effects, thus attenuating the inflammatory response [137]. On the other hand, exosomal miR-221 operates via the same mechanism. However, in this case, inhibiting this suppressor protein leads to alternative activation of the JAK/STAT pathway, through phosphorylation and activation of STAT1, thereby mediating M1 polarization [138].

Several miRNAs promoting differentiation into the M2 phenotype have also been identified. Among them are miR-934, miR-222 and miR-301a-3p, which are expressed in colorectal cancer, ovarian cancer and pancreatic cancer, respectively [139–141]. These target the PI3K/Akt signalling while inhibiting PTEN, a tumour suppressor protein. Consequently, the expression of genes related to M2 phenotype, such as VEGF, increases [139, 142, 143]. Other miRNAs such as miR-19b-3p and miR-29a-3p, target the JAK/STAT pathway, activating STAT3 and STAT6, respectively. The latter does so through the inhibition of SOCS1, thus enhancing M2 polarization [144–146].

Given the rising interest in therapies targeting exosomes and the recognition of exosomal miRNAs’ involvement in modulating immune responses and tumour progression within the TME, there is considerable value in deeper exploration of their identification and understanding of their mechanisms of action related to these functions. Such insights will undoubtedly enrich the development and refinement of targeted strategies leveraging this biological mechanism against TAMs, thereby paving the way for novel avenues in personalized cancer treatments.

### Epigenetic modifications

The epigenetic level, that is, changes that result in gene expression variations without altering the genetic sequence, are also tightly involved in the intricate regulatory machinery of macrophage polarization. These modifications include DNA methylation and histone modifications [136, 147].

DNA methylation involves the addition of a methyl group to a specific position on DNA by DNA methyltransferases (DNMTs), leading to the silencing of the target gene. Studies have shown that changes in the expression of certain DNMTs in macrophages are associated with the modulation of their phenotypes in the context of cancer [147–149]. For example, elevated levels of DNMT1 have been associated with an augmentation in M2 macrophages. In a preclinical investigation focusing on lung cancer, it was observed that estrogen administration triggers the upregulation of DNMT1 expression. Consequently, this leads to hypermethylation of the TP53 promoter, negatively impacting both its activity and p53 expression. Such alterations are linked with a poorer prognosis for the disease [150]. In another investigation, it was revealed that M2 TAMs heightened their DNMT1 expression via the IL-6-pSTAT3-ZEB1-DNMT1 axis within the TME of breast cancer, and were identified as pivotal contributors to tumour cell migration [151]. Similarly, another study has shown that exosomes derived from M2 macrophages promote proliferation and migration of tumour cells by increasing the expression of DNMT3A, a long non-coding RNA (lncRNA) called LINC00470, and myc. Microvesicles regulate the methylation of the miR-199a-3p promoter through the myc/DNMT3a axis mediated by LINC00470 [149].

The methylation and acetylation of histones have distinct impacts on macrophage regulation depending on their localization. For instance, the demethylation of histone 3-lysine 27 by Jumonji domain-containing protein D3 at the gene promoter region increases the expression of previously epigenetically silenced M2-related genes [152]. On the other hand, it is known that specific histone deacetylases (HDACs), such as HDAC3, HDAC6, HDAC7 and HDAC9 facilitate polarization towards M1 [153, 154]. Conversely, other HDACs such as SIRT1, SIRT2, and SIRT6 have been identified to promote M2 polarization by increasing the expression of anti-inflammatory cytokines [155, 156].

### Metabolism and autophagy

The plasticity of macrophages relies on metabolic changes that align with the specific requirements of the detected microenvironment. Depending on the energy demand for macrophage functions, different metabolic pathways adapt accordingly [157]. M1 macrophages primarily rely on increased glycolytic activity, fatty acid synthesis (FAS) [158], and the pentose phosphate pathway (PPP) [159]. Additionally, they exhibit alterations in the tricarboxylic acid cycle, leading to the accumulation of itaconate and succinate, resulting in HIF-1α stabilization [159, 160]. This enhances the expression of genes supporting glycolytic metabolism in this cell subtype. Furthermore, they express iNOS to produce NO from arginine, a key mediator in inflammation regulation [158, 161, 162]. M2 macrophages also heavily rely on high glycolytic activity and exhibit high lactate secretion similar to the M1 phenotype. However, they are associated with mitochondrial oxidative phosphorylation, fatty acid synthesis (FAS), and glutamine metabolism, accompanied by decreased activity in the PPP. In this phenotype, arginine is metabolized by Arg-1. It is important to highlight that the proper functioning of certain enzymes is crucial for regulating this process. For instance, inhibiting hexokinase, a key enzyme in the glycolytic process, has been shown to effectively decrease M1 polarization and consequently the secretion of proinflammatory cytokines [157]. Other notable enzymes include pyruvate kinase M2 isotype, which is associated with M1 orientation, and pyruvate dehydrogenase kinase, which promotes the M2 type. Additionally, the enzyme carbohydrate kinase-like protein, which inhibits the PPP, is highly expressed in M2-like macrophages, acting as a deterrent to M1 polarization [163].

There is increasing evidence that autophagy also plays a significant role in relation to macrophages [164]. Autophagy is a self-degrading system essential for maintaining cellular balance and energy supply, especially during critical stages of development and in response to perceived stress conditions in the microenvironment. It has been demonstrated that deficient autophagy in macrophages favours M1 polarization. Deletions of autophagy-related genes, such as ATG5 and Rnbcn, enhance M1-characteristics and the pro-inflammatory immune response [165, 166]. Conversely, increased autophagy activity by macrophages has the opposite effect of promoting M2-polarization. New experimental trials are being carried out to demonstrate that there is a direct relationship between autophagy and polarization towards an M2 phenotype [167]. Progress in unravelling the connection between metabolism regulation and the differentiation of these immune cells presents abundant opportunities for developing targeted therapies from an immuno-metabolic standpoint. Moreover, as the intricate relationship between autophagy and TAM modulation becomes increasingly apparent, a promising avenue emerges for effectively influencing this process. This advancement holds immense potential for revolutionizing our approach to cancer treatment.

## TAMs as key regulators of oncogenic processes and tumour progression

As the focus on understanding the pivotal role of TAMs in tumour development intensifies, it has become increasingly clear that TAMs exert a wide range of functions in shaping the TME in favour of tumour initiation and progression [93]. This underscores the significance of TAMs as pivotal participants throughout the entire spectrum of this pathology, involving various components of the immune system. However, it is important to note that TAMs are a heterogeneous population with diverse functions that can vary depending on the tumour type, stage, and location. Therefore, a better understanding of the mechanisms that regulate TAM-cancer cell interactions within the tumour niche is crucial for the development of effective cancer therapies. In this chapter, a detailed description of the diverse array of TAMs’ functions within the tumour progress will be provided.

### Tumour growth and angiogenesis

**Fig. 4:**
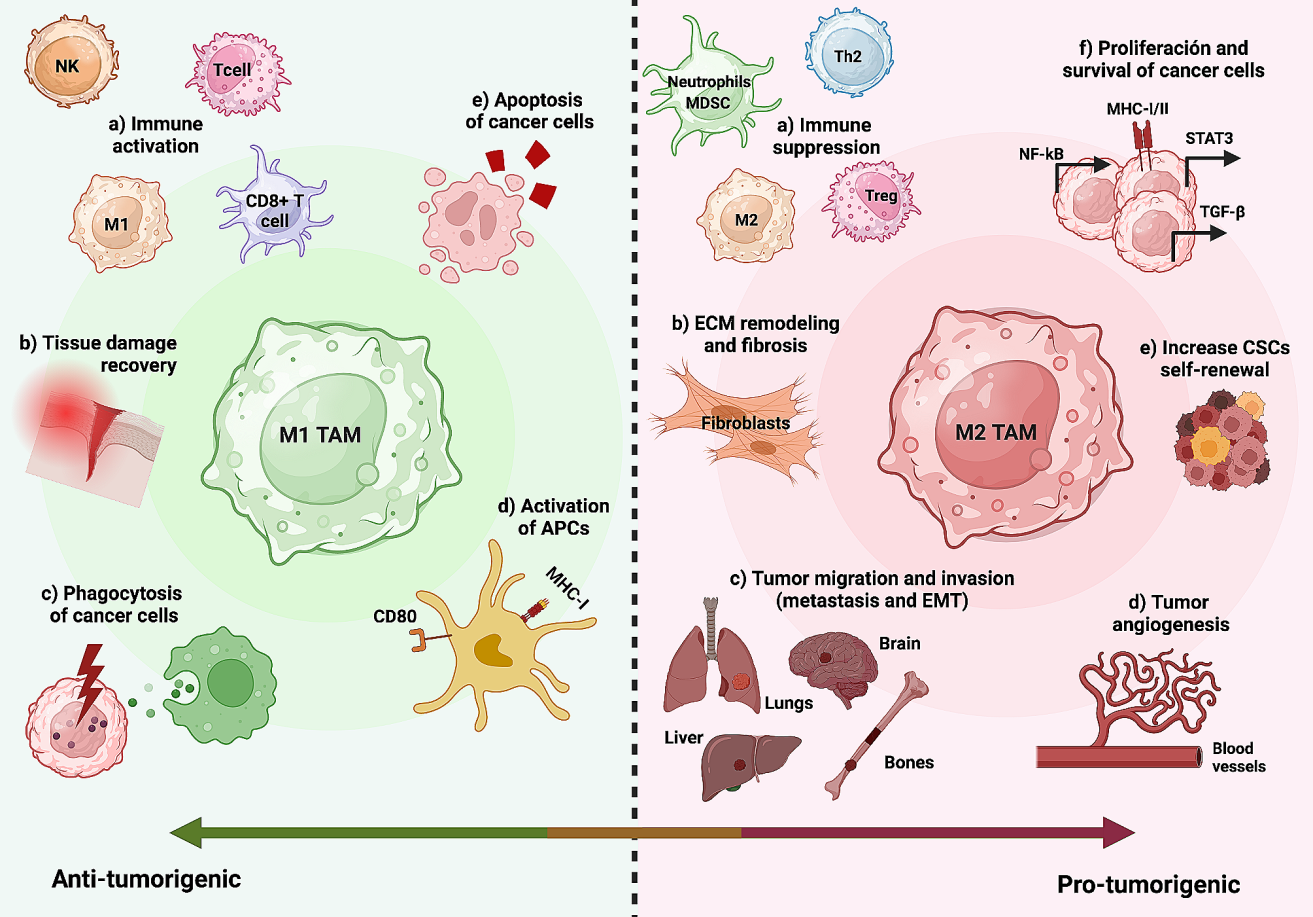
Tumour-associated macrophages (TAMs) are involved in various anti- and pro-oncogenic processes. A key characteristic of macrophages is their intrinsic plasticity, the two extremes of which have been identified as M1-type and M2-type polarization. Depending on the paracrine signals they receive as well as the type of tissue, microenvironment and stage of the tumour, they may lead to one phenotype or another. On the left side of the figure, anti-tumoural M1-like macrophages are depicted. These macrophages contribute to T cell recruitment and immune activation, particularly by stimulating NK cells. They exhibit direct cytotoxic and phagocytic effects on tumour cells. Additionally, M1-like macrophages aid in tissue repair, promote the maturation of antigen-presenting cells (APCs) necessary for efficient antigen presentation, and actively induce apoptosis in cancer cells. On the right side of the figure, pro-tumoural M2-like macrophages are illustrated. These macrophages, conditioned by the hypoxic TME, release immunosuppressive mediators, support tumour proliferation, angiogenesis, invasion, and metastasis. They induce epithelial-mesenchymal-transition, facilitate tissue remodeling and inflammation, and enhance the self-renewal rate of CSCs

It has long been established that angiogenesis and neovascularization play indispensable roles in tumour progression and metastasis. This requirement for constant nutrient supply arises due to the rapid proliferation of cancer cells, leading to the fast growth of the tumour mass [199]. Neovasculature from the primary tumour are defined by its tortuous and leaky characteristics. As a result, neighbouring areas around the tumour site are of hypoxic nature. This drives the expression of hypoxia-related genes as the transcription factors HIF-1/2α are stabilized and translocated to the nucleus for transcription of related genes [200]. This results in the tumour release of inflammatory factors such as IL-4 and IL-10, that attract TAMs to the tumour site and favour them to adopt a M2-like phenotype (Table 1). Werno and colleagues showed that knocking out HIF-1α in macrophages co-cultured in tumour spheroids resulted in reduced levels of CD31-positive cells, which is a marker for endothelial cell differentiation, a necessary condition for angiogenesis, hence indicating the indispensable role of HIF-1α in the process of TAM-mediated angiogenesis [201]. Upon TAMs’ recruitment to the tumour site and subsequent M2-like polarization, M2-like TAMs initiate an “angiogenesis cascade”, which involves the release of pro-angiogenic factors such as TNF-α that activates the NF-kB pathway after binding to its receptors TNFR1/2. In turn, NF-kB further promotes the growth and survival of cancer cells by controlling the synthesis of target genes, including VEGF, EGF, hepatocyte growth factor (HGF) and platelet-derived growth factor (PDGF), which in turn induces neo-angiogenesis [3] (Fig. 4). Moreover, TAMs release chemokines such as CXCL12 in a HIF-1-dependent manner which attracts CXCR-4-expressing endothelial cells, further aiding the process of neo-angiogenesis at the tumour site [200]. Interestingly, TAMs have also been found to contribute to the formation of endothelial tubular network at the new vessel branching site through the close collaboration with recruited endothelial cells, hence showing that TAMs are not only actively involved in initiation of neo-angiogenesis but also the structural conformation remodelling of the newly formed vessels [98].

The formation of new vessels is regulated by the growth factors released by cells in the TME, such as VEGF, basic fibroblast growth factor (bFGF), MMPs, PDGF, and angiopoietin-1 [202, 203]. The primary sources of PDGF, which causes pericyte infiltration, are TAMs and platelets [204]. Specifically, Tie-2 receptor-expressing monocytes, a subset of peripheral blood monocytes, are implicated as producers of proangiogenic TAMs [205]. Notably, vascular permeability is influenced by vessel maturation and remodelling, which is dependent on the interaction between pericytes and ECs [206]. When the Tie-2 receptor on ECs binds with the angiopoietin-1 produced from pericytes, the cell-cell junctions of ECs’ tighten, contributing to the stabilization of the newly created vasculature [207].

### TAMs and inflammation

In a physiological context, inflammation is initiated to restore balance in response to a disturbance caused by external factors [208]. However, prolonged inflammation increases the risk of developing into a conducive environment for cancer. Long before a tumour forms, inflammation can be induced, fostering tumour development through processes such as neo-angiogenesis, immune suppression, and the occurrence of oncogenic mutations [128].

As previously mentioned, M1-like TAMs have the ability to secrete proinflammatory factors such as TNFα and IL-1β in pancreatic cancer, IL-6 in breast cancer, and CXCL8 in endometrial cancer, which can lead to tumour-promoting inflammation [80, 209–212] (Fig. 4). Although this proinflammatory characteristic is expected to bolster an effective immune response against tumours, the notable plasticity of TAMs frequently links them with immunosuppression. The pro-inflammatory activity of IL-6, which is mediated by the JAK/STAT3 pathway, facilitates cell proliferation, differentiation, and death [128, 213]. The proinflammatory cytokine IL-1 induces ECs to produce VEGF, promoting angiogenesis and increases tumour invasiveness and metastasis [214, 215]. TAMs may thereby promote the development and spread of tumours through their characteristic inflammatory properties, especially in the case of persistent low-grade inflammation [216].

### TAMs and the extracellular matrix / tissue remodelling

Remodelling of tumour stromal components is another significant means by which a tumour-promoting microenvironment is fostered. In the process of tissue remodelling, TAMs closely collaborate with CAFs. CAFs secrete factors such as CXCL14 and chitinase 3-like 1 (Chi3L1) in breast cancer, which not only recruit macrophages to the tumour site but also induce their polarization toward the M2-like phenotype [217]. In turn, these M2-like TAMs secrete proteolytic enzymes, such as MMPs and cathepsins, that degrade the ECM and digests the basement membrane as well as the collagen surrounding the tumour [218]. Hagemann and colleagues showed that when TAMs are co-cultured with tumour cells, the expression of MMP2/7/9 are enhanced in a TNF-α-dependent manner, consequently promoting ECM degradation [219]. This degradation facilitates breast tumour cells to invade surrounding tissues and facilitates their dissemination to distant organs. In addition, TAMs are also tightly involved in the stiffening of the stroma surrounding the tumour by introducing crosslinks between collagen fibres in the ECM. This in turn promotes tumour progression and metastasis by mechanical forces [220] (Fig. 4). Maller and colleagues found that TAMs promote breast cancer progression through stroma stiffening by the expression of the cross-linking enzyme lysyl-hydroxylase 2 (LH-2). LH-2 induces the formation of hydroxylysine aldehyde-derived collagen crosslinks and the stromal expression of this enzyme was found to be strongly associated with disease-specific mortality, providing evidence that LH-2 can serve as a stromal prognostic biomarker [221].

Upon the degradation of basement membrane and resulting collagen fragments, chemotactic stimuli are further released which can attract more TAMs, hence further enhancing the re-arrangement of the ECM in a positive feedback mechanism, thereby preparing for processes like angiogenesis and tumour metastasis [222, 223]. Fascinatingly, TAMs have also been shown to enhance the expression and organisation of collagen I and VI by its deposition around the ECM surrounding the tumour tissue. This deposition serves as the physical barrier protecting the cancerous tissue from external assaults [224].

### The role of TAMs in tissue invasion and distant metastasis

Metastasis, the spread of cancer cells to distant organs, is the main cause of cancer-related deaths [1]. This colonization of different organs by cancer cells is a multistep process in which TAMs play a pivotal role [118]. The first stages of metastases are significantly influenced by the epithelial-mesenchymal transition (EMT) program, which is initiated after activation of TLR-4 on the surface of M2-like macrophages (Fig. 4). This program results in epithelial cells losing their cell-cell junction and acquiring a motile and invasive mesenchymal cell phenotype. Secreted cytokines from TAMs, such as IL-1, IL-6, TNF-α and TGF-β, promote EMT [225–227] (Table 2). Gao and colleagues showed that the inhibition of IL-6 prevented the increase of EMT markers such as vimentin and β-catenin in an STAT3/ERK-dependent manner, and in turn resulted in reduced cell migration and invasion in colon carcinoma cells [225]. Moreover, through the interaction of α4 integrin with vascular cell adhesion molecule-1 (VCAM-1) on the surface of cancer cells, TAMs boost the survival of cancer cells in circulation. Through this connection, the pro-apoptotic effects of molecules like TRAIL are inhibited in cancer cells by activating the PI3K/Akt survival pathway [228]. In fact, as tumour cells breach the extracellular membrane layer of ECs via MMPs and cathepsins released by TAMs, and enter the bloodstream, they are observed to directly interact with TAMs [118, 229]. Such interaction between tumour cells and macrophages enhances the process of extravasation.

Before metastasis occurs, there is a massive inflow of macrophages into the healthy tissue. Various molecules secreted by tumour cells, including TNF-α, VEGF, CSF-1, CCL-2, or TGF-β, attract macrophages to the bloodstream and prompt their aggregation at pre-metastatic locations [230]. In particular, CCL2-triggered chemokine cascade has been found to be actively involved in the promotion of pulmonary metastasis of breast cancer cells by enhancing the recruitment of metastasis-associated macrophages [231].

Moreover, macrophages that develop at potential metastatic sites facilitate the invasion of cancer cells by modifying collagen fibres, providing pathways for cancer cells migration [232]. Consequently, by releasing growth factors that have been deposited in the ECM, TAMs modify it and stimulate neo-angiogenesis, extravasation, and EMT [52]. This has been shown by Kim and colleagues where they performed a co-culture of tumour cells with or without pre-invaded macrophages into the ECM. Indeed, in the presence of pre-invaded macrophages the number of invading tumour cells and invasion distance are higher [232]. Moreover, it was found that as pre-invaded macrophages extravasate through the endothelium, they leave behind a “microtrack” along its migration route in the collagen matrix through realigning and degrading the collagen fibres as they form the invadopodia. This track then allowed the invasive tumour cells to follow along as it migrated through the ECM [232].

### TAMs and immune modulation

The balance between TAMs pro-inflammatory (M1-like) and anti-inflammatory (M2-like) phenotypes determines the immune response within the TME. As mentioned previously, M1-like TAMs promote anti-tumour immune responses, while M2-like TAMs suppress immune surveillance and promote tumour growth [119] (Fig. 4). M2-like TAMs have the capacity to suppress the activation of cytotoxic T cells, which are responsible for eliminating tumour cells, while M1-like TAMs promote the recruitment of immunosuppressive cells, including regulatory T cells, contributing to the establishment of an immunosuppressive TME [51, 233, 234].

Another mechanism through which TAMs promote immunosuppression and tumour progression is by releasing immunomodulatory factors such as PGE2, IL-8, IL-10 and TGF-β [235] (Table 2). IL-10 is crucial for maintaining tissue homeostasis during infections and inflammation by upregulating innate immunity, limiting excessive inflammatory responses, and promoting tissue repair mechanisms [236]. In a cancer setting, these exhibit their effects on other immune cells, such as T cells and NK cells by inhibiting their activity and promoting tumour cell survival through evasion of immune surveillance [15, 89]. It has been found that TAMs play a dual role in affecting the cytolytic activity of NK cells. On the one hand, TAMs impair NK cells’ cytolytic activity in a contact-dependent manner that is largely mediated through TGF-β. Upon inhibition of TGF-β, the cytotoxicity of NK cells was then shown to be restored. On the other hand, TAMs also induce NK cells exhaustion by promoting the CD27lowCD11high NK cell phenotype, which is characterised by its reduced activation and tumour killing abilities [237]. TAMs also play a role in the transformation of T-helper (Th) cells into Tregs through activation of Foxp3 in response to TGF-β secretion [132, 238, 239]. CD8 + effector T lymphocyte activity is then suppressed by Tregs that are induced by TAMs through the release of TGF-β and IL-10. Additionally, immunosuppression of CD8 + cytotoxic T cells is further aggravated by TAMs’ secretion of CCL5, CCL17, CCL20, and CCL22 chemokines, which attract CCR4 + Tregs and aid its infiltration into the TME [240, 241] (Table 2).

Further, as mentioned earlier, there is a well-documented positive interaction between TAMs and Tregs. Tregs play a role in promoting immune evasion in cancer by facilitating the establishment of an immunosuppressive TME. TAMs have been shown to activate Tregs, thereby promoting the differentiation of monocytes towards an M2 phenotype [242]. In a clinical trial, it was demonstrated that infiltration of M2-like immunosuppressive TAMs at metastatic sites inhibits clinically relevant immune responses in the metastatic TME (M-TME). In this study, the transcriptomic profile of 24 paired primary and metastatic tumour samples from patients with high-grade serous carcinomas (HGSCs) who did not receive neoadjuvant chemotherapy was compared. The analysis identified several genes involved in cytokine/chemokine signalling (such as IL-10 and CCL22) by M2-like TAMs as potential drivers of T-cell exhaustion in the M-TME. Patients with HGSC exhibiting robust M-TME infiltration by M2-like TAMs showed inhibited immune responses at metastatic sites, correlating with poor disease outcome. Moreover, 1468 genes were differentially expressed in the primary-TME versus M-TME of HGSCs, and infiltration by immune effector cells had little impact on patient survival [243].

Moreover, TGF-β and IL-10 released by TAMs have also been shown to have an effect on DCs. These anti-inflammatory cytokines not only impair proper DCs’ maturation and functioning, but also have been shown to induce apoptosis of these antigen-presenting cells, consequently resulting in reduced DCs’ infiltration in tumour metastasized sites, as well as reduced migration to lymph nodes, hence dampened T cell-mediated adaptive immune responses [244–246].

Direct interactions between TAMs and other immune cells can mediate another mechanism of immune response inhibition. Programmed cell death protein (PD-1), belonging to the CD28 superfamily, is a key component in immunosuppression. Considering PD-1 is crucial when modulating the immune system for various purposes such as combating cancers, preventing infections, addressing autoimmune disorders, and ensuring the survival of organ transplant recipients [247]. The ligand for PD-1, programmed cell death-ligand 1 (PD-L1), is produced by antigen-presenting cells. When antigen-presenting cells unite with T cell-carried PD-1, the combination prevents T cells from attacking [248]. The PD-1/L1 signalling pathway can restrict the activities of T effector cells, DCs and NK cells. This restriction increases the likelihood of tumour immune escape, as evidenced by the suppression of activation, proliferation and cytokine expression effects on T cells, and by the inhibition of the phagocytosis in TAMs [249–251] (Fig. 2).

By enhancing the expression of other surface proteins such as CD80/CD86, or death receptor ligands like Fas-L or TRAIL, TAMs can selectively suppress the immune response [252]. These ligands act as agonists for inhibitory receptors on immune effector cells, such as CTLA-4, FAS, and TRAIL-RI/-RII, respectively [253, 254]. When the PD-1 and CTLA-4 receptors are stimulated, the T cell receptor (TCR) signalling pathway is inhibited and the synthesis of cytokines and proteins that support cell survival is reduced. Additionally, TAMs generate the enzyme Arg-1, which breaks down L-arginine. L-arginine is essential for T cell-mediated antitumour response, TCR complex expression, lymphocyte proliferation, and the establishment of immunological memory [252, 255]. Thus, TAMs decrease immune responses in a pleiotropic manner, suppressing adaptive immunity against tumours.

Moreover, impaired immunological synapses in the process of macrophages antigen fragments presentation to T cells can lead to anergic and unresponsive T cells rather than activated [95, 256]. This T cell anergy status is maintained by the M2-induced Treg population, and is particularly observed in the tumour-draining lymph nodes [256, 257]. Furthermore, Kersten and colleagues (2022) showed that CD8 + T cells are primed to become exhausted upon prolonged, antigen-specific interaction with TAMs, and the exhaustion is particularly stimulated under a hypoxic environment as seen in the TME [258]. Moreover, tumour infiltration of CD8 + T cells is limited upon this long-lasting interaction with TAMs, hence resulting in an immune-excluded TME pattern. Peranzoni and colleagues showed that by applying CSF-1R blockade in combination with anti-PD-1 therapy, CD8 + T cell tumour infiltration was enhanced and tumour progression was delayed [259].

### M1-like TAMs and cancer cell elimination

M1-like TAMs have been demonstrated to be an effective means of eliminating cancer cells, despite the role of M2-like TAMs in the growth of tumours. M1-polarized macrophages can indeed act more effectively than M2-like macrophages, driving Th responses by antigen presentation, involving T cell proliferation and IFN-γ release [260]. IFN-γ-stimulated macrophages release proinflammatory cytokine IL-12, which has strong anticancer activity and the capacity to restore costimulatory qualities for T cells in TAMs [42, 113]. Furthermore, M1 polarization is driven by TLR ligands (like LPS) either by themselves or in conjunction with IFN-γ, which further inhibits the development of cancer cells [84] (Table 1). Lei and colleagues were able to generate a second generation of M1 macrophages developing a higher polarization rate and phagocytic capacity using the CAR technique, increasing their antitumour functions in different tumour models in vitro and in vivo, promoting the expression of IL-1 and IL-6, as well as apoptotic and cytolytic mechanisms of TAMs against tumour cells [261].

By producing cytokines and directing cytotoxicity towards tumour, activated M1-like macrophages play a protective role against cancer in the body (Fig. 4). The cytotoxicity of macrophages can be heightened by introducing M-CSF and muramyl dipeptide to in vitro macrophage cultures or by employing specificity-induced activation methods. The stimulation of antimicrobial agents and pathogens has been demonstrated to effectively enhance the cytotoxicity of macrophages against specific bladder cancer cell lines. An illustration of this approach is the use of Bacillus Calmette-Guérin (BCG) in bladder cancer treatment [262]. Furthermore, data suggests that the elevated levels of TNF-α, IL-6, and IL-12 in the urine of patients with bladder cancer improved by BCG treatment may be associated with macrophage activity [262].

### TAMs in self-renewal

A subset of cancer cells known as cancer stem cells (CSCs) have the capacity to proliferate and give rise to cancerous offspring. Because of their resistance to chemotherapy, CSCs have emerged as a key factor in the recurrence of tumours [263, 264]. Previous studies have indicated the positive reciprocal cross-regulation of CSCs and TAMs. It has been shown that when both CSCs and TAMs are introduced concurrently in syngeneic mouse models, there is an enhanced efficacy observed in tumour initiation and metastatic processes [265]. It has been documented that TAMs regulate CSCs’ ability to self-renew and their susceptibility to drugs by using an intricate web of cytokines, chemokines, growth factors, and ECM components (Fig. 4). According to a study by Yi and colleagues, glioma-initiating cells secreted more CCL2, CCL5, VEGF-A, and neurotensin than glioma cells did. These results imply that CSCs have a significant role in TAM recruitment by secreting macrophage chemoattractants [266].

TAMs have also been linked to different functions in CSCs self-renewal through the paracrine loop of the EGF signalling pathway [267]. Specifically, Yang and colleagues showed that TAMs activated the EGF signalling pathway in murine breast cancer cells, promoting CSC-like characteristics. The EGF signal was found to be essential for maintaining a CSC phenotype by promoting STAT3 phosphorylation and Sox-2 expression [267]. TME matrix elements can also alter TAM activity to promote CSC self-renewal. Hyaluronic acid (HA) generated by metastatic breast CSCs facilitated tumour invasion and metastasis into the bone microenvironment, as reported by Okuda and colleagues [268]. Mechanistically, HA promotes TAM-CSC contact, which is followed by TAM secretion of PDGF-BB. Then, via inducing the production of fibroblast growth factor FGF-7 and FGF-9, the TAM-derived PDGF-BB stimulates fibroblasts and bone stromal cells such as osteoblasts to facilitate CSC self-renewal [268]. Other contact-dependent interactions between TAMs and CSCs that drive tumour stemness include the binding of LSECtin on TAMs and BTN3A3 receptor on TAMs, as well as the binding of CD90 and Ephrin type A on CSCs with its corresponding ligands on TAMs [265, 269]. Collectively, these juxtacrine interactions facilitates and sustains the reciprocal relationship between CSCs and TAMs, promoting the survival of each other’s populations by establishing a tumour-permissive milieu.

## Current macrophage-based clinical interventions

The use of macrophages as a therapeutic target in cancer has emerged as a promising approach to combat tumour progression [53, 276].

**Fig. 5:**
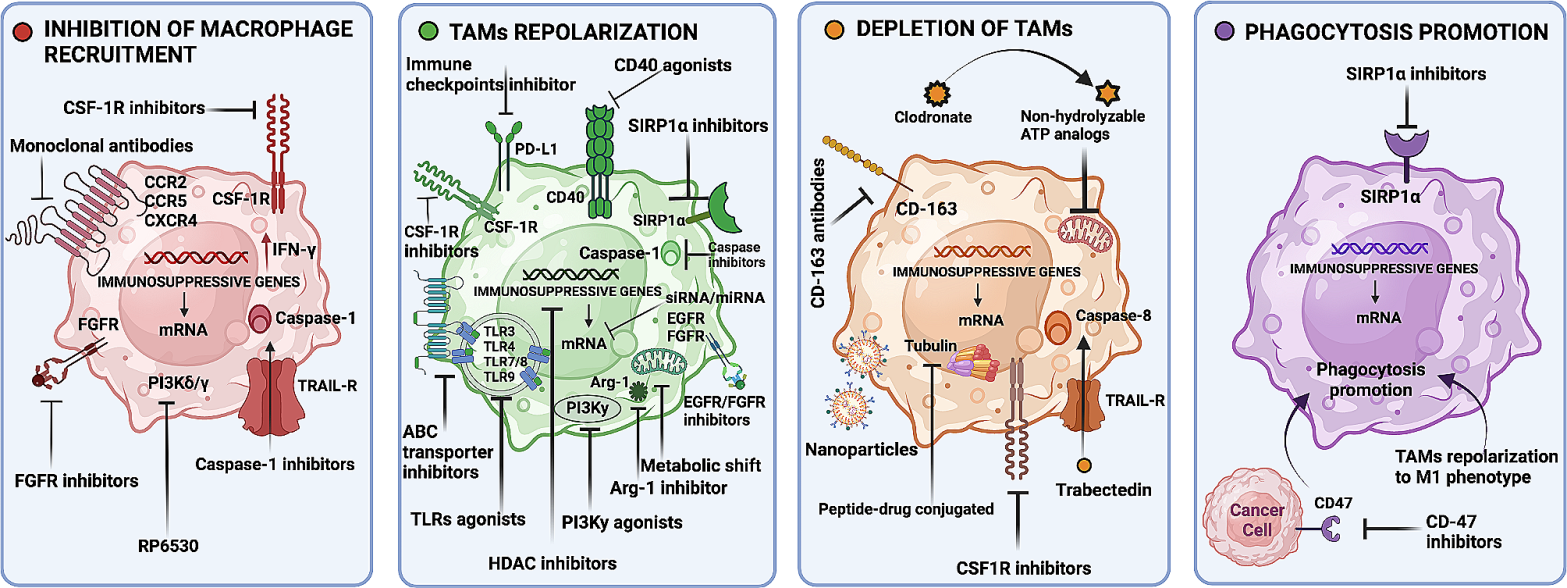
TAM-targeting treatment approaches. An overview of the most promising strategies to combat tumour progression by targeting TAMs in cancer therapy. The approaches are categorized into inhibition of macrophage recruitment (red), repolarization of TAMs (green), depletion of TAMs (yellow), or promotion of phagocytosis (purple)

Currently, advanced therapies targeting TAMs utilize diverse strategies aimed at controlling TAM abundance, phenotype, and functional state within the TME, as well as enhancing their capacity to combat cancer cells through various mechanisms. These approaches encompass a range of interventions, including inhibiting TAM recruitment, inducing TAM repolarization, depleting TAMs from the TME, and enhancing TAM phagocytic activity to promote an anti-tumoural phenotype (explained in Fig. 5). These multifaceted strategies collectively aim to disrupt the pro-tumourigenic functions of TAMs and harness their potential anti-tumour properties for therapeutic benefit. Current macrophage-based clinical interventions are summarized in Table 3.

**Table 3:** Macrophage-based clinical interventions

Therapy	Mechanism of action	Tumor type	Clinical Trial	Phase	Study Status
**Inhibition of Macrophage Recruitment**					
Carlumab (CNTO 888)	CCL2 blockade, to inhibit angiogenesis, macrophage infiltration and tumor invasion, which favor tumor growth and metastasis	Prostate cancer	NCT00992186	Phase II clinical trial [279]	Completed
Emepticap pegol (mNOX-E36)	CCL2 inhibition and therefore, of TAMs recruitment. In addition, it improves antiangiogenic treatment	GBM	-	Preclinical phase [281]	-
FOLFIRINOX + PF-04136309	CCR2 inhibition	Pancreatic neoplasms	NCT01413022	Phase I clinical trial [282]	Completed
BMS-813,160 + Chemotherapy or Nivolumab	CCR2-CCL2 axis blockade by antagonists	Pancreatic and colorectal cancer PDAC	NCT03184870 NCT03767582	Phase Ib/II clinical trial [283]	Completed Ongoing
Pexidartinib (PLX3397) + paclitaxel	CSF-1R inhibition	Solid tumors	NCT01525602	Phase Ib clinical trial [288]	Completed
Pexidartinib (PLX3397)	CSF-1R inhibition	TGCT, EAC	NCT04488822	Phase III clinical trial [289]; Preclinical phase [361]	Ongoing
Emactuzumab	CSF1R activation inhibition, leading to the recruitment of CSF1R-expressing macrophages that constitute the tumor mass	TGCT	NCT05417789	Phase III clinical trial [290]	Ongoing
BL-8040 + Pembrolizumab	CXCR4 inhibition and PD-1 blockade	Pancreatic cancer	NCT02907099	Phase IIb clinical trial [293]	Completed
LY2510924 + Durvalumab	CXCR4 inhibition and PD-1 blockade	Solid tumors	NCT02737072	Phase Ia clinical trial [294]	Completed
PF-06747143 with and without chemotherapy	CXCR4 inhibition	Acute myeloid leukemia	NCT02954653	Phase I clinical trial [295]	Completed
Tinengotinib (TT-00420)	CSF-1R inhibition	Breast cancer	-	Preclinical phase [362]	-
Plerixafor	CXCR4 inhibition	Colorectal ovarian pancreatic cancer	NCT02179970 NCT03277209	Phase I clinical trial [no results]	Completed
BMS-936,564 + lenalidomide/ dexamethasone or bortezomib/ dexamethasone	CXCR4 inhibition	MM	NCT01359657	Phase Ib clinical trial [363]	Completed
**TAMs repolarization**					
3D185	FGFR1/2/3 inhibition and CSF-1R kinase activity	Bladder breast and urothelial cancer, MM, NSCLC, LSCC, GA	-	Preclinical phase [297]	-
Pexidartinib (PLX3397)	CSF-1R inhibition	OS FS PDAC	-	Preclinical phase [298, 302]	-
MGN1703	TLR9 agonist	Small-cell lung cancer	NCT02200081	Phase II clinical trial [306]	Completed
GSK1795091	TLR4 agonist	Neoplasms	NCT03447314	Phase I clinical trial [307]	Completed
LHC165 or LHC165 + PDR001	TLR7 agonist	Solid tumors	NCT03301896	Phase I/Ib clinical trial [308]	Completed
RRX-001	NLRP3 inhibition, inducer of Nrf2 and NO superagonist	Small cell lung cancer	NCT03699956	Phase II clinical trial [311]	Completed
Metformin	Reduces oxidative phosphorylation and increases glycolysis	OS	-	Preclinicalphase [314]	-
2-desoxiglucose (2DG)	hexokinase inhibition	Pancreatic cancer	-	Preclinicalphase [315]	-
NCX-4016	NO release	Colon carcinoma and hepatoma	-	Preclinicalphase [316]	-
Tenalisib (RP6530)	Inhibits PI3Kδ/γ	T cell lymphoma Hodgkin lymphoma	NCT02017613	Phase I/II clinical trial [317] Preclinical phase [318]	Completed
Genetic deletion of ABC transporters	Cholesterol efflux blockage	Metastatic ovarian cancer	-	Preclinical phase [319]	-
IFN-γ	Induction of NOS2 transcription, which increases NO production in cancer cells, leading them to apoptosis and ferroptosis	Hepatoma Melanoma Colorectal cancer RCC	-	Preclinical phase [320]	-
L-norvaline	ARG-1 inhibition	Breast cancer	-	Preclinical phase [321]	-
Manganese- Albumin Nanocomplex	Activation of TLR4	Melanoma	-	Preclinical phase [323]	-
CART-mRNA complexes	Positive regulation of proinflammatory cytokines	Melanoma and B cell lymphoma	-	Preclinical phase [324]	-
Dimannose- functionalized biodegradable polymeric NPs containing mRNA	Phosphorylate and activate IRF5 to increase the expression of M1 genes and decrease the expression of M2 genes	Ovarian, melanoma cancer and GBM	-	Preclinical phase [325]	-
VEGF and PIGF siRNA	VEGF and PIGF inhibition	Breast cancer	-	Preclinical phase [328]	-
Tenalisib (RP6530) + romidepsin	Inhibits PI3Kδ/γ	T cell lymphoma	NCT03770000	Phase I/II clinical trial [364]	Completed
**Depletion of M2-like TAMs**					
Pexidartinib (PLX3397)	CSF-1R inhibition	Colorectal cancer and mesothelioma	-	Preclinical phase [259]	-
Melittin derived peptide-drug conjugate (M-DM1)	Inhibition of tubulin polymerization by inducing M2 macrophage apoptosis	Melanoma	-	Preclinical phase [330]	-
Pembrolizumab (MK-3475)	PD-1 blockade	Melanoma NSCLC	NCT01295827	Phase I clinical trial [332]	Completed
Zoledronic acid + doxorubicin	Macrophage depletion and increased anti-tumor activity of doxirubicin	Lewis lung carcinoma	-	Preclinical phase [335]	-
Zoledronate (Bisphosphonate)	Induction of apoptosis of TAMs and suppresses the release of proangiogenic	Breast cancer	NCT02347163	Phase II clinical trial [336]	Completed
Lipid NPs loaded with doxorubicin	Inmunosupresion of CD163 + TAMs	Melanoma	-	Preclinical phase [340]	-
Clo-LipoDOTAP	Disruption of the mitochondrial respiratory chain, cytotoxicity against M2	Melanoma	-	Preclinical phase [341]	-
Trabectedin	Targets TRAIL-R1/2, inducing direct caspase-8 dependent apoptosis	CLL	-	Preclinical phase [344]	-
Trabectedin	Targets TRAIL-R1/2, inducing direct caspase-8 dependent apoptosis	Melanoma, pancreatic and metastatic prostate tumour	-	Preclinical phase [345–347]	-
Trabectedin + Ipilimumab + Nivolumab	Targets TRAIL-R1/2, inducing apoptosis. PD-1 and CTLA-4 receptor inhibition	Sarcoma	NCT03138161	Phase I/II clinical trial [348]	Ongoing
Combination of nivolumab- ipilimumab + lurbinectedin	Induction apoptosis, inhibition the production of inflammatory/growth factors (CCL2, CXCL8 and VEGF) and affected monocyte adhesion and migration capacity	Small cell lung cancer	NCT04610658	Phase I/II clinical trial [no results]	Completed
Lurbinectedin + Atezolizumab	Induction apoptosis and PD-1 blockade	Small cell lung cancer	NCT04253145	Phase I/II clinical trial [no results]	Ongoing
Erlotinib derivative (TD-92)	Potentiates the antitumor effect of anti-PD-1 and reduces TAMs by down-regulation of CSF-1R	NSCLC	-	Preclinical phase [349]	-
Emactuzumab + Atezolizumab	Inhibition of CSF-1R	Metastatic solid tumors	NCT02323191	Phase I clinical trial [350]	Completed
**Promoting phagocytosis of TAMs**					
MiR-340	CD47 suppression	Pancreatic cancer	-	Preclinical phase [85]	-
Anti-CD47 blocking mAbs	Blockade of the interaction between CD47-SIRP-α	Colon carcinoma	-	Preclinical phase [354]	-
PEP-20 polypeptide	Blockade of CD47 and its interaction with SIRPα	Colon carcinoma, melanoma	-	Preclinical phase [355]	-
HU5F9-G4 cytokine	Enhancement of macrophage phagocytosis by blocking CD47	Solid tumors	NCT02216409	Phase I clinical trial [356]	Completed
Humanized AB21	Anti-SIRPα antibody, targets SIRPα to promote the phagocytosis of TAMs	Colon carcinoma, breast cancer and Burkitt lymphoma	-	Preclinical phase [357]	-
TTA-Q6 + RRX-001	TTA-Q6 disrupts calcium uptake by cancer cells, activating calreticulin on their surface and triggering immune responses. RRX-001 reduces CD47 protein, preventing cancer cells from evading the immune system	Lung cancer	-	Preclinical phase [358]	-
HBPdC (palladium-based bioorthogonal nanoplatform)	ROS production and M1 macrophage polarization. Reduction of CD47 promoting phagocytosis. Activation of sequestered prodrugs by bioorthogonal catalysis, allowing chemotherapy	Breast cancer	-	Preclinical phase [360]	-

*Abbreviations* AML, acute myelogenous leukemia; CCL2, cytokine (C-C motif) ligand 22; CCR2, cytokine (C-C motif) receptor 2; CLL, chronic lymphocytic leukemia; CSF-1, colony stimulating factor 1; CSF-1R, colony-stimulating factor-1 receptor; DLBCL, diffuse large B-cell leukemia; EAC, esophageal adenocarcinoma; EGF1, epidermal growth factor 1; FGFR, fibroblast growth factor receptor; FL, follicular lymphoma; FS, fibrosarcoma; GA, gastric adenocarcinoma; GBM, glioblastoma multiforme; IFN-γ, interferon-gamma; LSCC, lung squamous cell carcinoma; MM, multiple myeloma; NLRP3, NOD-like receptor family pyrin domain containing 3; NO, nitric oxide; NOS2, nitric oxide synthase 2; NPs, nanoparticles; Nrf2, nuclear factor erythroid 2-related factor 2; NSCLC, non-small cell lung cancer; OS, osteosarcoma; PDAC, pancreatic ductal adenocarcinoma; PI3Kδ/γ, phosphoinositide 3-kinase delta/gamma; PIGF, placental growth factor; RCC, renal cell carcinoma; ROS, reactive oxygen species; TAMs, tumor associated macrophages; TGCT, tenosynovial giant cell tumor; TLR, toll-like receptor; TME, tumor microenvironment; VEGF, vascular endothelial growth factor

### Inhibition of macrophage recruitment

TAMs are recognized for their recruitment to the TME and their potential contributions to immune suppression, angiogenesis, and metastasis [277]. Here, we will describe relevant strategies that have been developed to impede macrophage recruitment to the TME by blocking chemokine ligand receptors.

To begin with, the chemokine ligand CCL2 and its receptor CCR2 are implicated in the initiation and progression of various types of cancers. The CCL2-CCR2 signalling axis plays a crucial role in the recruitment of inhibitory immune cells. Interrupting the recruitment of monocytes to tumours and retaining monocytes in the bone marrow reduces the number of M2-like TAMs at primary and metastatic sites, which increases CD8 + T cells, ultimately inhibiting tumour growth and invasion. Therefore, CCL2 and CCR2 have become potential therapeutic targets for cancer treatment [278–280].

Preclinical studies have indicated that the suppression of CCL2 could significantly enhance the effectiveness of anti-angiogenic treatment in glioblastoma by inhibiting the recruitment of macrophages dependent on this ligand. Utilizing a CCL2 inhibitor, mNOX-E36, researchers successfully suppressed the recruitment of TAMs in a rat model of GBM expressing CCL2. Their findings demonstrated that inhibition of CCL2 led to a reduction in tumour volume [281].

Interestingly, these findings have also been corroborated by clinical studies. In a Phase I clinical trial (*NCT01413022*), the combination of the CCR2 inhibitor PF-04136309 with FOLFIRINOX was shown to be safe and tolerable in patients with borderline resectable or locally advanced PDAC. Furthermore, this trial determined the optimal Phase II dosage for PF-04136309 to be 500 mg administered twice daily [282]. Another study investigated the efficacy of the IgG1κ mAb carlumab (NCT00992186), targeting CCL2, in metastatic castration-resistant prostate cancer patients. Among the 46 patients enrolled in this Phase II trial, one patient achieved stable disease (SD) for over 6 months, and fourteen patients retained SD for more than 3 months. The median OS was 10.2 months. Despite promising preclinical data suggesting potential clinical benefits, carlumab failed to effectively block the CCL2/CCR2 axis or demonstrate significant antitumour activity as a standalone treatment [279].

Another significant chemokine receptor found on myeloid and TME infiltrating T-cells is CCR5. Alongside CCR2, it has been shown to facilitate the migration of monocytes and myeloid-derived suppressor cells [283]. In this context, a Phase Ib/II trial (NCT03184870), conducted without randomization and in an open-label manner, investigated BMS-813,160, a dual antagonist targeting CCR2/5, administered either alone or in combination with chemotherapy or nivolumab in individuals diagnosed with advanced pancreatic or colorectal cancer. Although results have not yet been published, the study is expected to assess safety, tolerability, objective response rate, median duration of response, and PFS. Secondary objectives include the pharmacokinetics and pharmacodynamics of BMS-813,160 and the immunogenicity of nivolumab.

Additionally, an ongoing single-center, two-arm Phase I/II trial (NCT03767582) sought to enhance immune responses by administering GVAX and nivolumab while concurrently inhibiting immunosuppressive tumour-associated macrophages through CCR2/5 inhibition with BMS-813,160. Preliminary findings indicate that this combination is safe, and the neoadjuvant application does not result in a surgical delay [284].

Additional example involves interfering with the CSF-1/CSF-1R axis, which is closely associated with the accumulation and migration of TAMs. Indeed, numerous studies are underway investigating drugs targeting this pathway for the treatment of breast, ovarian or gastric cancer with some currently in clinical trials [285–287]. In this context, the antibody pexidartinib in combination with paclitaxel was investigated in a completed Phase Ib study involving patients with advanced solid tumours (NCT01525602). The study demonstrated that this combination was well tolerated, establishing the recommended Phase II dose of pexidartinib to be 1600 mg/day. Of the 38 patients eligible for evaluating treatment efficacy, one achieved a complete response (CR), five had a partial response (PR), thirteen had SD, and seventeen experienced progressive disease (PD). Additionally, plasma CSF-1 levels increased by over 50-fold, indicating the efficient blockade of CSF-1/CSF-1R interactions by pexidartinib [288].

In another randomized Phase III study, the efficacy and safety of pexidartinib in patients with tenosynovial giant cell tumour were investigated (NCT04488822). The trial demonstrated the clinical benefit of pexidartinib, showing a significant tumour response compared to patients receiving a placebo. Notably, this study represents a groundbreaking achievement, as it marks the debut of a systemic therapy demonstrating such effects against this disease [289].

Additionally, the efficacy of emactuzumab, another humanized monoclonal antibody targeting CSF-1R, is being evaluated in the Phase III study NCT05417789 among subjects with tenosynovial giant cell tumour. However, since the trial began in 2024, no results have been posted yet [290].

The CXCL12/CXCR4 axis has also been implicated in monocyte recruitment and their subsequent differentiation into TAMs [4, 291]. In a breast cancer model, the production of CXCL12 by tumour cells resulted in increased blood vessel density and a higher influx of macrophages, thereby enhancing tumour cell invasiveness. Inhibition of CXCR4 with the antagonist AMD3100 resulted in the suppression of metastasis and tumour cell dissemination [292]. Numerous clinical trials are underway to assess the effectiveness of various CXCR4 antagonists. For instance, trials such as *NCT02179970* and *NCT03277209* have investigated the effects of the CXCR4 antagonist, plerixafor, on the TME and the safety of continuous intravenous administration in patients with solid tumours. However, to date, no results from these studies have been reported.

Additional studies are also being conducted with other CXCR4 antagonists. For example, the Phase IIb trial NCT02907099 evaluated the safety, efficacy, and immunological effects of the CXCR4 antagonist BL-8040 (motixafortide) in metastatic PDAC patients receiving pembrolizumab and chemotherapy [293]. Among the 22 patients receiving the combination treatment, the objective response rate, disease control rate, and median duration of response were 32%, 77%, and 7.8 months, respectively. Furthermore, BL-8040 was observed to enhance the infiltration of CD8 + effector T cells into the tumour while reducing immunosuppressive populations of MDSCs and regulatory Tregs. Overall, the trial suggested that the combination of CXCR4 and PD-1 blockade demonstrates additive effects in enhancing the responses of metastatic PDAC patients’ to chemotherapy.

In addition, a Phase Ia trial (NCT02737072), aimed to assess the safety and tolerability of the CXCR4 peptide antagonist LY2510924 in combination with durvalumab in subjects with advanced refractory solid tumours. Among the nine patients enrolled, the majority, including those with pancreatic or rectal cancer, completed one or two cycles of LY2510924 and durvalumab without any dose-limiting toxicities reported. Furthermore, four patients (44.4%) showed the best objective response, and one patient had an unconfirmed partial response. This indicated that the combination of LY2510924 with durvalumab was safe and well-tolerated in these patients [294].

On the other hand, the Phase I clinical trial NCT02954653 aimed at investigating a new CXCR4 antagonist IgG1 antibody, PF-06747143, in patients with acute myeloid leukemia was terminated prematurely, due to strategic business reasons, before reaching pharmacokinetics assessments [295].

However, inhibiting the infiltration of TAMs into the TME could thwart their protumorigenic activities and diminish the supportive network fuelling tumour progression. Additional research and refinement of this therapeutic approach are essential to specifically target the recruitment of M2-like TAMs in the future.

### TAMs repolarization

The phenotype and function of TAMs are determined by the stimulation of various extracellular factors [296]. When TAMs are recruited to the tumour zone and exposed to various cytokines in the TME, they undergo a gradual shift from an M1 to an M2 phenotype. This shift implies immune suppression and contributes to tumour progression. Therefore, reversing the phenotype of M2-type TAMs to M1-type TAMs could represent a novel and effective anticancer strategy [93].

Currently, several therapies are targeting the inhibition of the CSF-1R to induce reprogramming of TAMs in different types of tumours, including breast, bladder, multiple myeloma, lung, and osteosarcoma [297, 298]. CSF-1R serves as a survival factor for normal macrophages/microglia development. In studies involving mouse models of GBM, the inhibition of CSF-1R, has been found to reduce tumour progression, even without depleting TAMs [299, 300]. Moreover, a study by Tan and colleagues demonstrated in vivo that CSF-1R inhibition with PLX5622 (CSF-1R inhibitor) could reduce a subset of TAMs, increase the percentage of infiltrating cytotoxic T cells, decrease tumour volume and extend mouse survival within four weeks of treatment [301]. Mitchem and colleagues conducted an experiment in which a combination of anti–CSF-1 antibody (clone 5a1) and a small-molecule inhibitor of CSF-1R kinase (PLX3397) was administered to mouse models with pancreatic cancer. The results revealed a significant enhancement in the response to chemotherapy, accompanied by increased infiltration of CD8 + T cells into the tumour of these mice. This indicated that CSF-1R inhibition not only suppressed the tumourigenicity of pancreatic cancer cells but also, as observed for mammary tumours, induced chemosensitivity and recruited CD8 + T-cells. In another similar study, treatment with PLX3397 simultaneously reduced TAMs and regulatory T cells and increased CD8 + T cell infiltration in the microenvironments of primary and metastatic osteosarcoma sites [298, 302]. There are also drugs such as 3D185, showing promising results as antitumour candidates. This drug significantly inhibits the kinase activity of FGFR1/2/3 and CSF-1R, thereby suppressing FGFR signalling and tumour cell growth in both in vitro and in vivo models. Additionally, it can inhibit macrophage survival and M2-like polarization, reversing the immunosuppressive effect of macrophages on CD8 + T cells. Furthermore, it inhibits FGFR3-induced cancer cell migration of CSF1-differentiated macrophages [297].

There is also a significant focus on studying TLRs as a potential target for restoring the M1 phenotype. These receptors regulate the reprogramming of TAMs. Specifically, TLR3, TLR4, TLR7/8, and TLR9 are known to enhance the immunological control of malignant diseases, converting M2-type TAMs into the M1 phenotype thereby limiting tumour progression [303–305]. Currently, clinical trials involving TLR agonists for tumour therapy are underway. For instance, a completed Phase II study examined the TLR9 agonist MGN1703 in small-cell lung cancer patients after receiving the first-line chemotherapy cycles (*NCT02200081*) [306]. Despite showing a promising safety profile, no significant differences in OS and PFS were observed between patients receiving MGN1703 (twice-weekly, 60 mg s.c.) and the controls receiving the local standard of care. Another completed Phase I study investigated the combination of intravenously administered TLR4 agonist GSK1795091 with other immunotherapies, including pembrolizumab, in 54 patients with advanced solid tumours (*NCT03447314*) although no conclusions could be made regarding this compound’s anti-tumour activity due to the limited data collected [307]. Another completed Phase I study from 2021 explored the effect of a TLR7 agonist, LHC165 (*NCT03301896*) administered intratumourally as a single agent (*n* = 20), and in combination with the investigational PD-1 inhibitor spartalizumab (PDR001) (*n* = 19) in patients with advanced solid tumours. Overall, the combination treatment was shown to be safe and tolerable among patients [308].

The metabolism of M2-type TAMs is characterised by increased glycolysis and decreased oxidative phosphorylation. This metabolic shift is known as the Warburg effect, and is commonly associated with cancer cells [309, 310]. Interestingly, recent studies have shown that targeting the metabolism of M2-like TAMs can shift their polarization from a pro-tumoural phenotype to an anti-tumoural one, making them more effective in combating tumours [53]. Energy consumption is essential for recruitment, migration, and function of TAMs and there are already some clinical trials of drugs targeting their metabolism. For example, a Phase III study investigating the anticancer alkylating agent RRX-001 in patients with small cell lung cancer is underway (*NCT03699956*) [311]. RRX-001 is a small molecule immunotherapeutic that has been shown to inhibit the enzyme glucose 6-phosphate dehydrogenase (G6PD) in addition to also inhibiting c-Myc and downregulating CD47 [312]. Activation of this crucial enzyme involved in the pentose phosphate pathway has been shown to induce M2-like polarization through upregulating the release of M2-polarizing factors like CCL2 and TGF-β1 in triple-negative breast cancer cells [313].

Numerous examples of preclinical studies investigating TAMs’ repolarization can also be found in the literature. Metformin, a hypoglycemic medication, has shown in certain studies to reduce the M2-like polarization of TAMs in mice pancreatic tumour and bone sarcoid tumour models [314]. Although the molecular mechanism of 2-deoxyglucose’s ability to specifically block M2-TAM’s glycolysis in TME and eliminate its tumour-promoting phenotype is yet unknown, it is a competitive inhibitor of hexokinase 2, and has been shown to disrupt the pro-metastatic activities of M2-TAMs [315]. Moreover, active caspase-1 can promote TAMs to accumulate lipids and differentiate into a pro-tumourigenic phenotype. Inhibitors of this enzyme such as NCX-4016 have been shown to induce the rewiring of TAMs into an anti-tumourigenic phenotype and halt tumour growth in vivo [316]. PI3K is an important protein in cell signalling, and its δ and γ isoforms are predominantly expressed in hematopoietic origin cells. Hyperactivation of the PI3K/AKT pathway in macrophages has been identified to be involved in the pathogenesis of some types of cancer and also affects disease outcome. Modulation of this pathway by PI3Kδ/γ inhibitors such as RP6530, either as a single agent or in combination with another drug, is already being evaluated in clinical trials (*NCT02017613*, *NCT03770000*) in patients with T-Cell Lymphoma [317] and also through preclinical studies. A Phase Ib study showed that administering 800 mg of RP650 twice daily was deemed safe and tolerable in relapsed/refractory T-cell lymphoma patients, and the overall response rate in 35 patients was 45.7%, from which 3 had a CR, 13 PR, and the median duration of the response was 4.9 months [317]. he results of RP6530 are also very promising in Hodgkin lymphoma. Locatelli and colleagues showed that RP6530 negatively regulated lactic acid metabolism in Hodgkin lymphoma human xenografts, changing macrophage activation from an immunosuppressive M2-like phenotype to a more inflammatory M1-like state, resulting in reduced tumour angiogenesis and tumour regression [318]. On the other hand, tumour cells promote cholesterol efflux from the membrane of M2-like TAMs via an ATP-binding transporter (ABC transporter) to facilitate a more conducive environment for their growth and survival. Consequently, in mouse models of ovarian cancer, inhibiting membrane sterol efflux from M2-like TAMs through genetic deletion of the ABC transporter led to a transformation of the M2-TAM’s pro-tumour phenotype into an anti-tumour one [319]. This transporter, moreover, induces the production of IFN-γ production, suppressing genes associated with M2-like and activating M1-like TAM phenotype. In vitro studies of hepatoma, melanoma, colorectal cancer, and renal cell carcinoma have already been conducted, demonstrating the importance of IFN-γ-induced reprogrammed glycolytic metabolism in reducing tumour growth [320]. Other molecules that may have significant therapeutic implications in the context of TAM reprogramming are Arg-1 inhibitors. This enzyme drives activation towards the M2 phenotype. It has been demonstrated that L-norvaline suppressed overexpressed Arg-1 in M2-like TAMs in a mouse model of breast cancer, thus shifting the balance of arginine metabolism and reversing the phenotype [321].

Other important approaches that promote M1-type polarization involve using small molecules like micro-RNAs or nanoparticles (NPs) [320, 322, 323]. In one study, it was found that trace Mn2 + ions, bound to bovine serum albumin to form Mn-BSA nanocomplexes, stimulated pro-inflammatory responses in human- or murine-derived macrophages through TLR4-mediated signalling cascades. Furthermore, they observed that these nanocomplexes reprogram tumour-associated macrophages and inhibit the growth of melanoma tumours in vivo. Hence indicating these nanocomplexes as promising TLR4-agonists to be further studied in a clinical setting, particularly attributable to their superior biosafety than typical TLR4-agonists such as LPS [323]. In another study, a mixture of OX40L, CD80, and CD86-encoding mRNA was delivered intratumourally using charge-altering releasable transporters (CARTs). These CART-mRNA complexes effectively transfected tumour-infiltrating immune cells, including 28% of TAMs, and stimulated systemic anti-tumour immunity, ultimately curing tumours in both melanoma and B cell lymphoma models [324]. Following this line, Zhang and colleagues conducted a study in which they delivered biodegradable polymeric NPs functionalized with di-mannose moieties on their surface in mice models of ovarian cancer, melanoma, and GBM. These NPs contained two mRNAs aimed at directing and re-educate TAMs. The included mRNAs are encoded for IRF5 and IKKβ which are M1-polarizing transcription factors, to phosphorylate and activate IRF5. This, in turn, increased the expression of M1 genes such as CCL5, promoting an anti-tumour immunity, and decreased the expression of M2 genes such as Serpinb2 and CCL11 [325]. Furthermore, small interfering RNA (siRNA) have been employed to silence the expression of genes that control TAMs’ immunosuppressive properties [326, 327]. Mannosylated dual pH-responsive NPs loaded with two siRNAs targeting placental growth factor and VEGF were created by Song and colleagues. These two growth factors stimulate tumour development, angiogenesis and immunosuppression and they are overexpressed in TAMs, as well as breast and lung cancer cells. These NPs effectively transferred siRNAs to TAMs and cancer cells in a mouse breast cancer model, leading to gene silencing, suppression of tumour proliferation and metastasis, as well as, reversing the TME from pro-oncogenic to anti-tumoural [328]. All these findings support the therapeutic potential of reprogramming TAMs from multiple distinct aspects such as metabolism, signalling pathways, and targeting crucial M1/M2-polarizing factors to enhance cancer treatment outcomes.

### Depletion of M2-like TAMs

The most intuitive strategy to counteract the tumour-promoting effects of M2-like TAMs in immunotherapy is to eliminate them. Their elimination in clinical treatment can be effectively achieved by combining targeted immunotherapy, chemotherapy, and radiation. Various methods have been developed for this purpose, including antibody-mediated targeting of TAMs-specific markers, the use of chemotherapeutic drugs that selectively kill TAMs, and receptor-mediated depletion using NPs and other nanotechnologies [329]. Jeong and colleagues were able to specifically reduce M2-like TAMs in a mouse model of melanoma using M-DM1, which is a conjugation of melittin, as a carrier for M2-like TAMs, and mertansine, as a payload to induce apoptosis of TAMs. These results concluded with a suppression of tumour growth, migration and invasion, an improvement of the survival rates and an increase of the infiltration of CD8 + cytotoxic T cells and natural killer cells in the TME [330].

Studies have demonstrated that M2-like TAMs inhibit the function of CD8 + T cells by slowing their proliferation and blocking their activation through interaction with inhibitory immunological checkpoints [296, 331]. In colorectal cancer and mesothelioma mouse tumour models, the depletion of TAMs using the CSF-1R inhibitor PLX3397 led to the restoration of CD8 + cell infiltration and migration within the tumour islets and enhanced the effectiveness of anti-PD-1 immunotherapies [259]. A study conducted on melanoma and NSCLC patients receiving pembrolizumab (*NCT01295827*) revealed that a higher frequency of pre-existing intratumoural CD8 + T cells correlated with a positive clinical response to anti-PD-1 treatment [332].

An example of a cytotoxic drug coupled with nanotechnology is paclitaxel-loaded NPs. This approach has been efficiently applied in the clinic for the treatment of ovarian cancer, breast cancer and lung cancer inducing the depletion of CD68 + TAMs [333, 334]. Moreover, similar effects have also been shown with the combination treatment of liposomal doxorubicin (DOX) with zoledronic acid, a drug belonging to the class of bisphosphonates, exhibiting high affinity and toxicity to TAMs. Hattori and colleagues showed in mice bearing murine Lewis lung carcinoma that in addition to the TAM-depleting effects of zoledronic acid, the treatment also led to an increase in inflammatory cytokines including IL-12, GM-CSF and TNF-α, hence also indicating that an inflammatory response in the tumour tissue was induced by the zoledronic acid [335].

In contrast, a Phase II clinical trial evaluating the application of pre-operative zoledronate in triple negative breast cancer prematurely ended after 18 months because of a low accrual rate (*NCT02347163*) [336]. Further trials to assess the anti-tumour activity of zoledronic acid are currently ongoing in combination with other drugs and as an adjuvant therapy (*NCT03358017*, *NCT02595138*, *NCT04045522*). However, TAMs’ depletion may also have undesired consequences, such as impaired wound healing and compromised anti-infective immunity. Therefore, the identification of TAMs-specific targets for cancer therapy and cautious application of TAMs depletion strategies are necessary to minimize potential adverse effects.

Since macrophages play essential roles in erythropoiesis, homeostasis, and host defense, non-specific reduction of TAMs may be detrimental [337]. It has been demonstrated that M2-like macrophages and TAMs are marked by the scavenger receptor CD163, enhancing their pro-tumour actions in both mice and humans [338, 339]. Etzerodt and colleagues demonstrated in a mouse melanoma model that selective reduction of CD163 + macrophages re-educates the TME through enhanced recruitment of T cells and monocytes, both contributing to tumour regression. This was achieved using CD163 mAbs coupled with lipid NPs loaded with DOX. It is interesting to note that in this paradigm, the therapeutic benefits of specifically targeting CD163 + TAMs were nullified by pan-targeting of TAMs [340]. These results may help explain the general lack of effectiveness seen with treatment options currently undergoing clinical trials, such as CSF-1/CSF-1R or CCL2/CCR2 inhibition, which indiscriminately target all macrophages [32, 96, 288, 300]. Therefore, it is necessary to have a deeper comprehension of the particular TAM fraction that primarily drives tumour growth.

Another approach involves releasing clodronate and converting it into non-hydrolyzable ATP analogues that disrupt the mitochondrial respiratory chain, hence exhibiting cytotoxicity against M2-like TAMs since TAMs can phagocyte clodronate liposomes (Clo-LipoDOTAP) throughout the body. Interestingly, new formulations of clodronate liposomes have been created and have been shown to dramatically decrease the development of primary tumours and angiogenesis, and eliminate M2-like TAM in B16/F10 subcutaneous tumours [341]. Piaggio and colleagues showed that their novel Clo-LipoDOTAP inhibited growth, decreased viability and induced apoptosis in a macrophage-like cell line. Moreover, their liposomes significantly reduced the volume of primary tumours in melanoma-bearing mice [341].

TRAIL-R is a receptor belonging to the TNF receptor superfamily. TNF-related apoptosis-inducing ligand binds to TRAIL-R, inducing apoptosis in cancer cells or M2-like macrophages. This presents a promising target for cancer therapy as it selectively induces apoptosis in cancer cells while sparing normal cells. Furthermore, TRAIL-Rs expressed on neutrophils and lymphocytes differ from those expressed on TAMs and monocytes. Neutrophils and lymphocytes express the decoy TRAIL-R3, whereas monocytes and macrophages express the functional TRAIL-R1/2 [342]. This distinct characteristic has been exploited in the development of targeted drugs against TAMs, and one such registered therapeutic agent is Trabectedin [343]. Trabectedin, as proved in a preclinical investigation, induces leukemic cell death and depletes immuno-suppressive MDSCs and TAMs in a chronic lymphocytic leukemia mouse model [344]. Trabectedin’s anti-tumour efficacy has also been documented in pre-clinical models of melanoma, pancreatic tumour, and skeletal metastatic prostate tumour through its effects on TAMs [345–347]. This agent selectively targets TRAIL-R1/2 expressing cells, including TAMs, by inducing direct, caspase 8-dependent apoptosis in these cell populations. Currently, several ongoing clinical trials are investigating the effect of combinatorial immunotherapy, including Trabectedin [343]. A Phase I/II trial (*NCT03138161*) on advanced soft tissue sarcoma patients who received Ipilimumab, Nivolumab, and Trabectedin as first-line treatment recently concluded that the treatment is safe and effective (OS = 24.6 months and PFS = 6.7 months in Phase II with 79 participants), with no unexpected adverse events associated with triple therapy and maximum tolerated dose of Trabectedin determined to be 1.2 mg/m2 administered as continuous intravenous infusion over 24 h every 3 weeks. Hence, a Phase III trial of this combination is warranted [348]. Similar trials are also ongoing with a Trabectedin analogue, Lurbinectedin, an FDA-approved agent for the treatment against non-small cell lung cancer (*NCT04253145*) and others has been completed (*NCT04610658)* but in both cases no results from these studies have been reported.

Preclinical studies carried out by Shih and colleagues in tumour specimens from 22 NSCLC patients has found out that the combination of TD-92, a novel Erlotinib derivate which possesses anti-tumour effects across cancer cell lines, with anti-PD-1 resulted in a potent anti-tumour response in a Lewis lung carcinoma model, reducing the number of pro-tumourgenic TAMs as well as CSF-1R expression in macrophage cell lines, the reduction of tumour growth and a survival increased [349].

Another Phase Ib study (*NCT02323191*) evaluated the safety, antitumour activity as well we the pharmacokinetics and pharmacodynamics of mAb Emactuzumab in combination with PD-L1 blocking mAb Atezolizumab in 221 patients with advanced solid tumours. This combination seemed to develop a safety profile with a considerable objective response rate (ORR) and an increase of CD8 + tumour infiltrating T lymphocytes as well as a relative TAMs reduction [350].

However, the depletion of TAMs does not lead to sustained anti-cancer response. Thus, the anticancer effect is somewhat less effective compared to strategies that involve blocking TAMs recruitment and reprogramming TAMs.

### Promoting the phagocytosis capacity of TAMs

Enhancing the phagocytic capacity of TAMs is an innovative approach to cancer treatment. The immune checkpoint pathway expression of macrophages, CD47-SIRPα, is known to diminish TAMs’ ability to recognize and phagocytose tumour cells [351] (Fig. 2). CD47 is typically overexpressed in tumour cells, hindering TAMs from effectively recognizing and phagocytosing these tumour cells. SIRPα, a myeloid inhibitory receptor found in myeloid cells as the macrophages, binds to the CD47 ligand on the cell surface, thereby restricting innate immunity [352]. Preclinical models of human lymphoid tumours, bladder cancer, and breast demonstrate that anti-CD47 antibody treatment promotes adaptive immunity and exerts anti-tumour effects via CD8 + T cells and DCs [353]. Tseng and colleagues showed in their in vivo experiments that using anti-CD47 antibodies not only enables macrophages to engulf cancer cells but also stimulates an immune response from cytotoxic T-cells and decreases the presence of regulatory T-cells [354]. This suggests that targeting the CD47/SIRPα axis through immunotherapy could potentially enhance the anti-tumour response by activating both the innate immunity mediated by macrophages and the adaptive immunity of T-cells. Another interesting strategy involves the use of anti-CD47 peptides, as shown by Wang and colleagues [355]. Their novel peptide pep-20, bound to CD47 with high affinity, blocked the CD47/SIRPα interaction through reducing the tyrosine phosphorylation of SIRPα on macrophages. Moreover, they showed significant enhancement of macrophage phagocytosis in their co-culture containing macrophages and either solid or haematological tumour cells. Moreover, tumour growth inhibition was also achieved in tumour-bearing mice receiving pep-20, with no significant toxicity observed at a dose of 2 mg/kg daily for 14 days and exhibited inferior blood toxicity than the anti-mouse CD47 antibody that they used as their positive control. In addition, systemic administration of their more stable peptide pep-20-D12, increased CD8 + T cell population in tumour tissues as well as enhanced its anti-tumour activities as demonstrated by the elevated release of IFN-γ from tumour-infiltrating lymphocytes and draining lymph nodes. And more interestingly, the combination of pep-20-D12 with a single irradiation (20 Gy) in tumour-bearing mice resulted in delayed or even complete regression in tumour growth [355].

Certain miRNAs involved in the regulation of the CD47/SIRPα axis have also been investigated in the pre-clinical setting. Xi and colleagues identified miR-340 as a negative regulator of CD47 expression in PDAC cells [85]. Overexpression of miR-340 significantly decreased CD47 expression at both the mRNA and protein levels in PDAC cells. Similarly, a high miR-340 expression in tumour cells enhanced the macrophage-mediated phagocytosis of PDAC cells, which was further increased when CD47 was blocked with an antibody. Moreover, tumour volume and weight of mice inoculated with PDAC cells overexpressing miR-340 were significantly lower than mice inoculated with PDAC cells transfected with the control miRNA. Tumour-infiltrating immune cells were analysed from tumours of mice bearing the PDAC cells overexpressing miR-340 or the control miRNA. Interestingly, the ratio of M1/M2-like macrophages and CD8 + T cells’ population significantly increased in the miR-340-overexpression group [85]. Encouraging results are also seen in the clinical setting. A completed Phase I trial (*NCT02216409*) investigating the safety of a humanized IgG4 antibody that targets CD47, Hu5F9-G4 (5F9), in patients with advanced solid tumours concluded that 5FU was safe and tolerable at the priming dose of 1 mg/kg on day 1, followed by maintenance doses of up to 45 mg/kg weekly, hence Phase II trials are warranted [356]. Besides targeting CD47, antibodies directed against SIRPα have also been studied. Kuo and colleagues developed the humanized AB21 (hAB21), a pan-allelic anti-SIRPα antibody, which was shown to enhance macrophage-mediated antibody-dependent phagocytosis of tumour cells in a dose-dependent manner and irrespective of the SIRPα genotype [357]. Moreover, the combination of hAB21 with anti-PD-1 treatment significantly delayed tumour growth, resulting in complete regression of the tumour in 6 out of 10 mice with colorectal carcinoma, whereas none was seen in mice treated with either when given as a single agent. In addition, the combination treatment induced the activation of CD8 + DCs and monocytic DCs, as well as enhanced the anti-tumour activity of effector T cells as seen in the increase of IFN-γ from CD4 + and CD8 + T cells in the spleen, and granzyme B release from CD8 + T cells in the tumour [357].

Other interesting combinatorial approaches include the use of a calcium channel inhibitor (TTA-Q6) and CD47 inhibitor (RRX-001), as investigated by Guo and colleagues in lung tumour mice models [358]. Calreticulin, a lectin-like chaperone, moves to the cell surface as a result of inhibiting calcium uptake in tumour cells, which in turn aids in activating macrophages and inducing DC maturation. While the simultaneous administration of the CD47 inhibitor prevents tumour immune evasion as it enhances macrophage phagocytosis of tumour cells [358].

Another approach targeting the CD47/SIRPα axis include the use of antibody-drug conjugates (ADCs), which represent one of the most successful types of drug conjugates in current therapeutics. Si and colleagues investigated such ADC consisting of an anti-CD47 mAb coupled to the drug mertansine in triple-negative breast cancer cells [359]. It was found that this ADC could significantly inhibit tumour growth in breast cancer mice models and enhanced macrophage infiltration and phagocytosis in the TME. Also, an effective adaptive immune response was induced which involved a rise in CD69 + NK cells, CD11c + DCs and CD4 + T cells [359].

Finally, Feng and colleagues developed a novel gene-editable nanoplatform utilizing palladium-based bioorthogonal chemistry. This platform, named HBPdC, integrates CRISPR/Cas9 gene editing system-linked Pd nanoclusters with a hyaluronic acid surface layer. HBPdC effectively induces the generation of reactive oxygen species within the tumor microenvironment (TME) and promotes macrophage M1 polarization, leading to the elimination of tumor cells and augmentation of the antitumor response of macrophages. Moreover, HBPdC reprograms tumor cells by downregulating the expression of CD47, thereby facilitating their recognition and phagocytosis by macrophages. Additionally, HBPdC enables chemotherapy and enhances tumor cell death while demonstrating a high level of biocompatibility and biosafety. This innovative approach holds promise as an effective toolset for cancer therapy applications [360].

Enhancing the phagocytic capacity of TAMs in cancer can directly impede tumour growth and metastasis, while also amplifying the effectiveness of other anti-cancer treatments, such as chemotherapy and immunotherapy. Therefore, leveraging the phagocytic potential of TAMs emerges as a promising strategy to bolster the immune response against cancer and enhance patient outcomes.

## Conclusions and future prospects

TAMs play pivotal roles in tumour immunity and response to immunotherapy, exhibiting notable heterogeneity and multifaceted functions within the TME. Therefore, elucidating their precise regulatory mechanisms and identifying macrophage-specific targets are imperative to optimize current immunotherapeutic strategies.

The use of macrophages as a therapeutic target in cancer has emerged as a promising approach to combat tumour progression, but the overall results from the clinical trials may not be as optimistic as expected. In these lines current therapies targeting TAMs still need to overcome major issues, added to the substantial development and production costs associated with engineered macrophage therapies [276, 365, 366]. In this respect, while inducing TAM clearance appears logical to counteract TAMs’ tumour-supportive effects, a significant obstacle is the nonspecific depletion of monocytes, extending beyond targeting TAMs specifically [329, 340]. Thus, selective targeting of TAMs, while sparing tissue-resident macrophages, is critical to mitigate adverse effects and ensure treatment safety and efficacy. Further, approaches that include targeting inflammatory cytokines, blocking inhibitory receptors, enhancing antigen presentation, modulating metabolic pathways, and using immune checkpoint inhibitors to induce TAM exhaustion can lead to immune-related adverse reactions, with immune cells attacking normal tissues.

Another promising strategy involves inhibiting TAM recruitment to the TME, which has the potential to enhance immunotherapy efficacy. This approach relies on various strategies, including interfering with chemokine signalling and utilizing monoclonal antibodies or small molecule inhibitors. However, it’s crucial to carefully consider potential resistance mechanisms and adverse effects, such as accelerated metastasis, in future clinical studies.

Stimulating the phagocytic capacity of TAMs emerges as a promising technique that promises to eradicate a large number of the tumour cell population, thus favouring the results of chemo- and immunotherapy. Although there are still not enough studies on this technique, continuing to focus on CD47-SIRPα signalling and using macrophages as soldiers against tumour cells may have promising results in the coming studies.

Finally, polarization of TAMs towards an M1-like phenotype has also aroused the interest of the scientific community. While reducing M2 TAM abundance or promoting M2-to-M1 transformation could be crucial in tumour therapy, challenges persist in refining this approach due to poorly understood polarization mechanisms and unpredictable immune responses among patients. In fact, attributing the role of the “villains” solely to M2 TAMs is challenging. Some studies have questioned the role of M1s in promoting inflammation and cancer development. Moreover, the subtle distinction between one phenotype and another, coupled with the multitude of factors influencing plasticity between phenotypes, varying across cancer types and even among patients, complicates the definition of therapeutic strategies.

In summary, while treatment strategies focusing on macrophages in precision medicine hold promise, achieving an effective immunotherapy still requires a multidisciplinary approach. This involves developing combined therapies that comprehensively address the intricate mechanisms governing macrophage behaviour within the TME.

## Data Availability

No datasets were generated or analysed during the current study.

## Declarations

**Institutional review board statement** Not applicable.

## Abbreviations

ABC: Transporter ATP-binding transporter
ADCs: Antibody-drug Conjugates
AKT: Protein Kinase B
APCs: Antigen Presenting Cells
ApoE: Apolipoprotein E
Arg-1: Arginase-1
ABC transporter: ATP-binding transporter
BCG: Bacillus Calmette-Guérin
bFGF: Basic Fibroblast Growth Factor
CAFs: Cancer-associated Fibroblasts
CARTs: Charge-altering Releasable Transporters
CCL: Chemokine (C-C motif) ligand
CCR: Chemokine (C-C motif) receptor
Chi3L1: Chitinase 3-like 1
circRNA: Circular RNA
COX: Cyclooxygenase
CR: Complete response
CRT: Calreticulin
CSCs: Cancer Stem Cells
CSF-1: Colony-stimulating Factor-1
CSF-1: R Colony-stimulating Factor-1 Receptor
CXCL: C-X-C motif chemokine factor ligand
DCs: Dendritic Cells
DNMTs: DNA methyltransferases
DOX: Doxorubicin
ECM: Extracellular Matrix
EGF: Epidermal Growth Factor
EMT: Epithelial-Mesenchymal Transition
FAS: Fatty Acid Synthesis
FGF: Fibroblast Growth Factor
GC: Glucocorticoid
GBM: Glioblastoma
GM-CSF: Granulocyte Macrophage-Colony Stimulation Factor
HA: Hyaluronic acid
HSCs: Hematopoietic Stem Cell
HDACs: Histone deacetylases
HGSCs: High-grade Serous Carcinomas
HIF: Hypoxia-inducible Factor
IC: Immune Complex
IFN-γ: Interferon-gamma
IL: Interleukin
iNOS: Inducible Nitric Oxide Synthase
IRF: Interferon Regulatory Factor
ITIM: Immunoreceptor Tyrosine-based Inhibitory Motif
JAK/STAT: Janus kinase/signal transducer and activator of transcription
lncRNA: Long non-coding RNA
LPS: Lipopolysaccharide
mABs: Monoclonal Antibodies
M-CSF: Macrophage Colony-stimulating Factor
M-TME: Metastatic Tumour Microenvironment
MHC: Major Histocompatibility Complex
MDP: Muramyl Dipeptide
MDSCs: Myeloid-derived Suppressor Cells
MEK: Mitogen-activated Protein Kinase
miRNA: MicroRNA
MMPs: Matrix Metalloproteinases
mRNA: Messenger RNA
mTOR: Mammalian Target of Rapamycin
NF-kB: Nuclear Factor Kappa B
NK: Natural Killer Cells
NO: Nitric Oxide
NPs: Nanoparticles
NSCLC: Non-small cell lung cancer
OR: Overall Response
OS: Overall Survival
PAMPs: Pathogen-associated Molecular Patterns
PD-1: Programmed Cell Death Protein
PD-L1: Programmed Cell Death-ligand 1
PD: Progressive Disease
PDAC: Pancreatic Ductal Adenocarcinoma
PDGF: Platelet-derived Growth Factor
PFS: Progression-free Survival
PGE2: Prostaglandin E2
PI3K: Phosphatidylinositol 3-Kinase
PKC: Protein Kinase C
PPAR: Peroxisome Proliferator-activated Receptor
PPP: Pentose phosphate pathway
PR: Partial response
PrCR: Programmed Cell Removal
ROS: Reactive Oxygen Species
RNS: Reactive Nitrogen Species
SD: Stable Disease
Siglec: Sialic Acid Binding Ig-like Lectin
siRNA: Small Interfering RNA
SIRPα: Signal Regulatory Protein Alpha
SOCS: Suppressor of cytokine signalling
STAT3: Signal Transducer and Activator of Transcription 3
TAMs: Tumour-associated Macrophages
TANs: Tumour-associated Neutrophils
TCR: T Cell Receptor
TEMs: Tie-2 Receptor-expressing Monocytes
TGF-β: Transforming Growth Factor-beta
Th: T-helper cell
TLR: Toll-like Receptor
TME: Tumour Microenvironment
TNF-α: Tumour Necrosis Factor-alpha
TRAF: TNF Receptor-associated Factors
Tregs: Regulatory T cells
VCAM-1: Vascular Cell Adhesion Molecule-1
VEGF: Vascular Endothelial Growth Factor
